# HMIL@ZIF-67/SiC Composite with MOFs-on-MOFs Structure for Activated Persulfate Degradation of Ciprofloxacin

**DOI:** 10.1021/acsomega.6c04084

**Published:** 2026-10-06

**Authors:** Haonan Pei, Siyuan Liu, Jiaqi Yuan, Nannan Wang, Yongxin Lei, Qun Wei, Yanqiu Zhu

**Affiliations:** † State Key Laboratory of Featured Metal Materials and Life-cycle Safety for Composite Structures, MOE Key Laboratory of New Processing Technology for Nonferrous Metals and Materials, School of Resources, Environment and Materials, 12664 Guangxi University, Nanning 530004, China; ‡ Faculty of Environment, Science and Economy, 3286 University of Exeter, Exeter EX4 4QF, U.K.

## Abstract

To address the challenge of thoroughly removing the antibiotic ciprofloxacin (CIP) using conventional processes, this study employed a MOFs-on-MOFs strategy. Utilizing silicon carbide (SiC) as the electron transport medium, cobalt-based zeolite imidazolate framework (ZIF-67) was in situ formed on the surface of a titanium-based metal–organic framework (MIL-125). This approach successfully constructed the MIL@ZIF-67/SiC ternary heterojunction composite. Characterization revealed that this material significantly suppressed metal ion leaching by forming Ti–O–Co bonds, while the introduction of SiC effectively promoted interfacial charge transfer. Performance evaluation demonstrated that the optimized MIL@ZIF-67/SiC catalyst (1:2 ratio) achieved over 96% CIP degradation within 30 min, exhibiting broad-spectrum degradation capabilities toward pollutants including tetracycline and Rhodamine B. Mechanistic analyses suggest that the Co^2+^/Co^3+^ redox cycle in ZIF-67 plays a central role in PMS activation and reactive oxygen species generation. Titanium oxide clusters in MIL-125 regulate the electron density at cobalt sites via interfacial charge transfer, while the incorporation of SiC is proposed to facilitate interfacial electron transfer and promote the regeneration of Co^2+^ from Co^3+^. These results suggest that the synergistic interaction among MIL-125, ZIF-67, and SiC facilitates PMS activation and sustains the catalytic redox process. Density functional theory calculations were employed to identify the reactive sites of CIP molecules. This study provides new insights for designing multimetal MOF heterojunction environmental functional materials.

## Graphical abstract

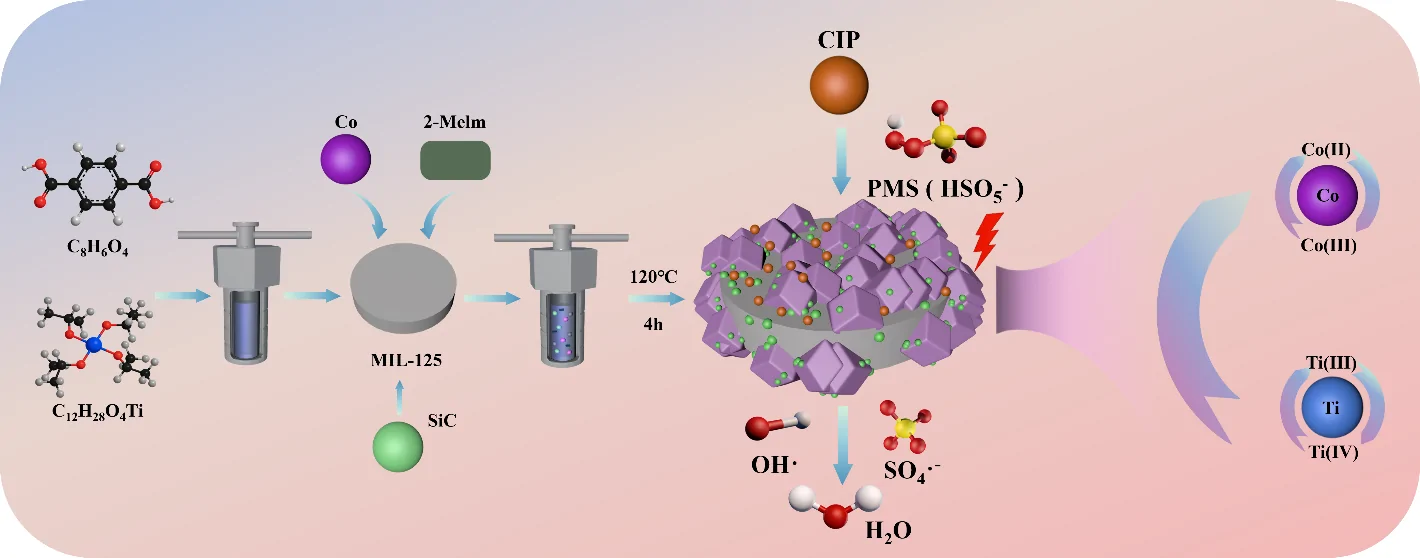

## 1 Introduction

The widespread use and improper disposal of antibiotics in human healthcare and livestock farming have led to their frequent detection in various aquatic environments, including surface water, groundwater, and wastewater from sewage treatment plants.^1^ They have emerged as a category of emerging pollutants of significant concern. Among these, ciprofloxacin (CIP) is a common fluoroquinolone antibiotic.^2–5^ , CIP possesses broad-spectrum antibacterial activity^6–8^ and is widely used in human medicine and livestock farming.^9^ However, its high consumption rate, stable chemical structure, and persistent ecological toxicity pose serious threats to aquatic environmental safety and human health.^10^ Nevertheless, CIP’s large molecular weight and complex structure^11,12^ limit the degradation efficiency of traditional wastewater treatment processes—such as adsorption and photocatalysis—which heavily depend on reaction conditions and require extended reaction times. Consequently, developing novel water treatment technologies capable of deeply and efficiently removing CIP has become particularly urgent. Currently, advanced oxidation processes (AOPs) have emerged as a promising technology for removing various organic and pharmaceutical pollutants from aqueous solutions.^13^ In AOPs, peroxymonosulfate (PMS) has garnered significant attention as a stable and effective oxidant.^14–16^ Upon activation, PMS decomposes to generate highly reactive species such as the sulfate radical (SO_4_^•–^), concurrently producing multiple other oxidizing species including hydroxyl radicals (·OH)^17,18^ and surface-activated complexes. These reactive species possess high oxidation potentials^19^ and relatively long half-lives, enabling them to rapidly and nonselectively attack most recalcitrant organic pollutants.^19^ Additionally, the half-life of SO_4_^2–^ can reach several microseconds to tens of microseconds, longer than that of ·OH, facilitating more thorough contact and reaction with pollutants in complex water bodies. Furthermore, PMS itself possesses an asymmetric structure with a relatively low O–O bond energy, making it more readily decomposed by various activation methods.^20^ Among various activation methods, the transition metal cobalt is considered the most effective activating component.^21–23^ However, Co still faces numerous challenges in practical water treatment applications. For instance, its chemical stability in aqueous solutions is insufficient, and its active sites are prone to agglomeration or dissolution during the catalytic process. This directly impacts catalytic efficiency and cycle stability,^24^ thereby limiting the practical implementation of AOPs to some extent. Therefore, developing highly efficient, stable, and reusable heterogeneous catalysts is crucial for advancing the application of this technology.

Among numerous heterogeneous catalysts, metal–organic frameworks (MOFs) demonstrate tremendous application potential due to their ultrahigh specific surface area, tunable pore structure, and abundant metal active sites.^24–27^ ZIF-67 is a framework material constructed with Co as the metal node and 2-methylimidazole as the ligand.^28,29^ It achieves efficient activation of PMS through its metal active center Co. However, ZIF-67 exhibits poor structural stability^30,31^ and the leaching of its transition metal can cause secondary pollution,^32^ presenting certain limitations during long-term use. Additionally, ZIF-67 has a high tendency to agglomerate,^33^ preventing the complete exposure of its active sites. According to reports, the construction of MOFs-on-MOFs composite structures offers an effective approach to addressing the aforementioned challenges.^34–36^ The heterostructure constructed by modifying the inner MOF substrate with an outer MOF layer not only effectively suppresses ZIF agglomeration but also exposes more accessible active sites, thereby enhancing the material’s overall catalytic activity. Furthermore, the interfacial coupling between different MOFs is expected to generate synergistic effects, promoting interfacial charge transfer^37^ and consequently improving the material’s stability and catalytic efficiency. Zhou et al.^38^ constructed PCN-222­(Fe)@UiO-67 to catalyze the epoxidation of alkenes for the degradation of organic substrates. Compared to either PCN-222­(Fe) or UiO-67 alone, this composite exhibited enhanced size selectivity toward substrates. Notably, it demonstrated negligible catalytic activity toward macromolecular substrates while efficiently converting small-molecule substrates, highlighting the synergistic effect of size-selective catalysis. This demonstrates that the MOFs-on-MOFs strategy offers novel insights for designing and developing high-performance heterogeneous catalysts. MIL-125 is a titanium-based MOFs synthesized via the sol–gel method. Its unique pancake-like structure provides a favorable surface for the dispersion and deposition of ZIF-67, thereby effectively preventing the agglomeration and loss of active sites. MIL-125 exhibits outstanding chemical^39^ and thermal stability.^40^ Its Ti–O metal cluster structure maintains excellent structural integrity in aqueous reaction environments, providing the structural foundation for the catalyst’s long-term use.^40^ Additionally, MIL-125 possesses a high specific surface area and abundant pore structure, which facilitates the adsorption and enrichment of reactant molecules. This enhances local concentrations, thereby accelerating reaction kinetics, and holds promise for generating synergistic catalytic effects with cobalt active sites.

Silicon carbide (SiC), as a typical ceramic material, exhibits excellent chemical stability, high thermal conductivity, and unique semiconductor properties.^41–43^ Its excellent electronic conductivity may facilitate rapid separation and transfer of interfacial charges during the catalytic process,^44^ thereby accelerating the activation cycle of PMS. Liu et al. constructed AE-SiC-modified ZIF-67 through a complex structural strategy, which increased the number of reaction sites and reduced the aggregation of ZIF-67 nanoparticles. This facilitated the rapid activation of PMS, thereby accelerating the degradation of pollutants.^45^ We speculate that the incorporation of SiC may synergize with the MOF components to collectively enhance the degradation efficiency of CIP.

Based on the aforementioned background and design, this study employed a MOFs-on-MOFs strategy to construct a surface-loaded catalyst. The MIL@ZIF-67/SiC composite material was designed and synthesized, and its performance in activating PMS for CIP degradation was systematically evaluated. The microstructure and crystallographic characteristics of the material were characterized using SEM, XRD, FT-IR, and other techniques. Experiments investigated the influence of key parameters on degradation performance, including catalyst dosage, PMS concentration, initial CIP concentration, solution pH, and reaction temperature. Combining radical quenching experiments and XPS analysis, the reaction mechanism was explored, revealing the generation pathways of active species and the pivotal role of covalent state cycling in PMS activation. Furthermore, cyclic degradation tests and coexisting ion impact assessments clarified the catalyst’s stability and applicability in real aquatic environments. This study provides a viable material design solution to address the challenge of balancing activity and stability in advanced oxidation applications using single MOF materials. It also offers theoretical foundations and experimental references for functionalizing MOF materials and developing highly efficient water treatment catalysts.

## 2 Experimental Section

### 2.1 Chemicals and Reagents

The chemicals used in this study are listed in Text S1.

### 2.2 Preparation of Catalysts

Preparation of MIL-125: MIL-125-NH_2_(Ti) was synthesized via a solvothermal method. Dissolve 0.13 g of NH_2_BDC in 3 mL methanol and 3 mL dimethylformamide, then add 100 μL of TTIP. After stirring at room temperature for 5 min, transfer the mixture to a stainless steel autoclave. Heat to 125 °C and maintain for 5 h, then cool to room temperature. Wash the product three times with dimethylformamide and methanol, respectively, followed by centrifugation. Vacuum dry the yellow product at 60 °C for 24 h.

Preparation of Etched SiC: Nano-SiC was prepared via the previously reported alkali etching method.^46^ This involved grinding silicon carbide powder in a vertical planetary ball mill at 400 rpm for 6 h, followed by sieving through a 350-mesh screen to obtain fine silicon carbide powder. Subsequently, 8 g of granular sodium hydroxide was dissolved in 100 mL of water, to which 4 g of SiC was added. The mixture was stirred at 500 rpm and heated to boiling for 2 h. The supernatant was then removed. Surface sodium hydroxide was eliminated using high-temperature ultrapure water. After cooling to room temperature, the mixture was centrifuged for 10 min and washed three times. It was subsequently washed with an ethanol solution and dried at 60 °C, yielding etched nano-SiC.

Preparation of MIL@ZIF-67/SiC: Disperse 0.12 g of synthesized MIL-125 powder and 0.2 g of polyvinylpyrrolidone (PVP) in 20 mL of anhydrous methanol. Sonicate to achieve complete dispersion and form a uniform suspension. Weigh different masses (0.182 g, 0.364 g, 0.546 g) of cobalt nitrate hexahydrate (Co­(NO_3_)_2_·6H_2_O) and add them to the above solution. Continuously stir magnetically at room temperature to form a uniformly mixed Solution A. Take the corresponding masses of 2-methylimidazole (0.205 g, 0.410 g, 0.615 g respectively) and sonicate to dissolve in 10 mL of anhydrous methanol, forming a clear, transparent Solution B. Under continuous magnetic stirring, slowly pour Solution B into Solution A. Subsequently, add 50 mg of silicon carbide (SiC) to this mixture and continue stirring. Transfer the mixture to a 50 mL high-pressure reactor lined with polytetrafluoroethylene (PTFE), seal it, and react in a forced-air drying oven at 120 °C for 4 h. After the reaction, allow the reactor to cool naturally to room temperature. Centrifuge and wash multiple times with anhydrous methanol and deionized water. Finally, the collected solid powder was dried overnight in a vacuum oven at 60 °C to obtain the final MIL@ZIF-67/SiC composite powder. The synthesis schematic is shown in Figure 1.

**Figure 1:**
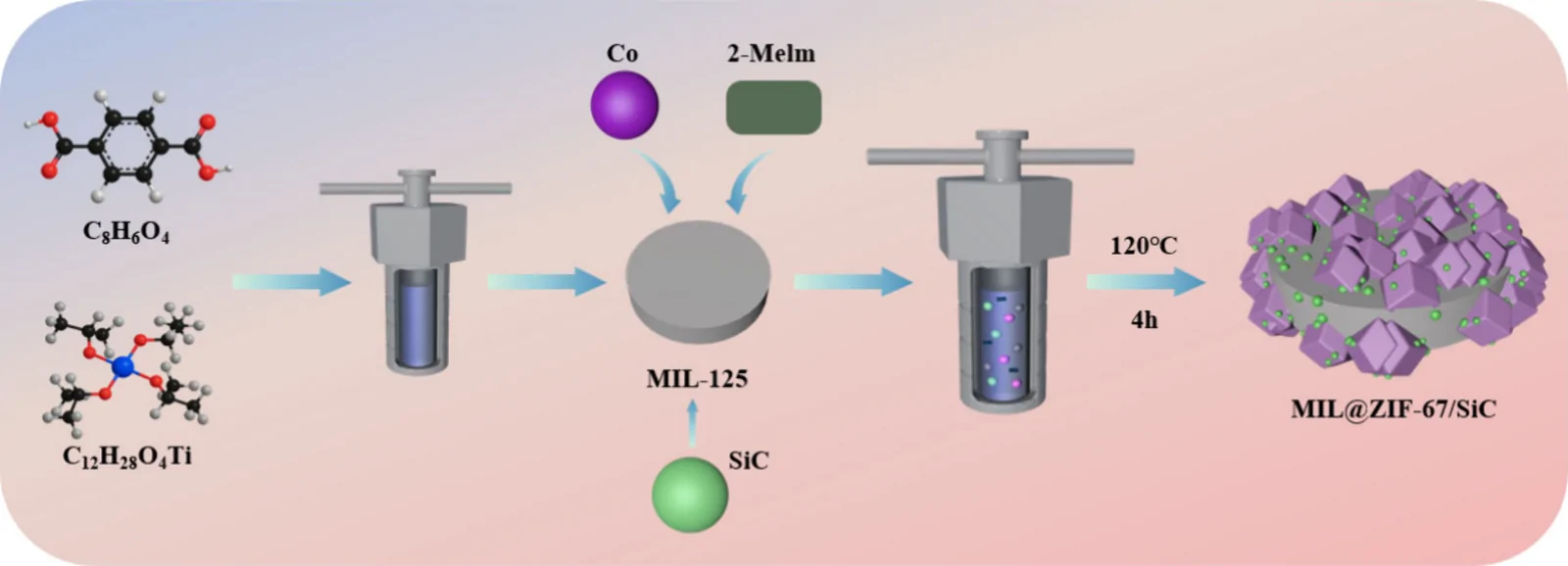
Schematic diagram of MIL@ZIF-67/SiC synthesis.

### 2.3 Characterizations

The characterization methods applied in this work were described in Text S2.

### 2.4 Procedures of Experiment

The CIP degradation experiment was conducted in a 250 mL beaker under stirring at room temperature. First, Ciprofloxacin Hydrochloride monohydrate was dissolved in deionized water and stirred continuously until complete dissolution. The solution was then transferred to a 1 L volumetric flask, diluted to volume with deionized water, and stirred for 12 h to obtain a CIP solution with an initial pH of 6.7 and a concentration of 20 mg/L. For the degradation experiment, a specific volume of the solution was poured into a beaker, and 4 mL was withdrawn as the −5 min sample. Subsequently, a quantified amount of catalyst was added to the remaining solution and sonicated for 5 min. Immediately after sonication, a sample was taken and designated as the 0 min reaction start sample. The beaker was then placed on a magnetic stirrer, and peroxymonosulfate (PMS) was added to initiate the degradation reaction. Reaction solutions were collected at 2, 4, 6, 8, 10, 15, and 20 min postinitiation. Each sample was immediately injected into a test tube containing 50 μL of sodium thiosulfate solution to quench free radicals and terminate the reaction process. All samples were filtered through a 0.22 μm PTFE membrane and measured for absorbance at 275 nm using a UV–visible spectrophotometer. CIP concentrations were calculated from a standard curve to analyze concentration changes during degradation.

The degradation rate and pseudo-first-order constant of the reaction are calculated using eqs 1 and 2.

1 $$\eta \left(\%\right)=\frac{C_{0}-C_{t}}{C_{0}}\times 100\%$$

2 $$k_{obs}\left(t\right)=\ln\frac{C_{0}}{C_{t}}$$

In eq 1, η (%) denotes the degradation rate, where *C*_*t*_ (mg/L) and *C*_*0*_ (mg/L) represent the CIP concentration at time t and the initial CIP concentration of the solution, respectively. In eq 2, *k*_*obs*_(*t*) denotes the pseudo–first–order rate constant, and *t* (min) represents the reaction time.

## 3 Results and Discussion

### 3.1 Morphological and Structural Analysis

Scanning electron microscopy (SEM) analysis of the sample surface is shown in Figure 2. Individual MIL-125 exhibits a classic pancake morphology with dimensions around 1 μm and relatively flat surfaces in Figure 2a, which provides favorable surface sites for the formation and deposition of ZIF-67. In Figure 2b, ZIF-67 shows the characteristic rhombic dodecahedral structure with well-defined morphology and uniform size, consistent with typical metal–organic framework crystal features. Figure 2c depicts the morphology of etched SiC, exhibiting a granular structure with surface roughness. After etching, the particle size was approximately 80 nm.

**Figure 2:**
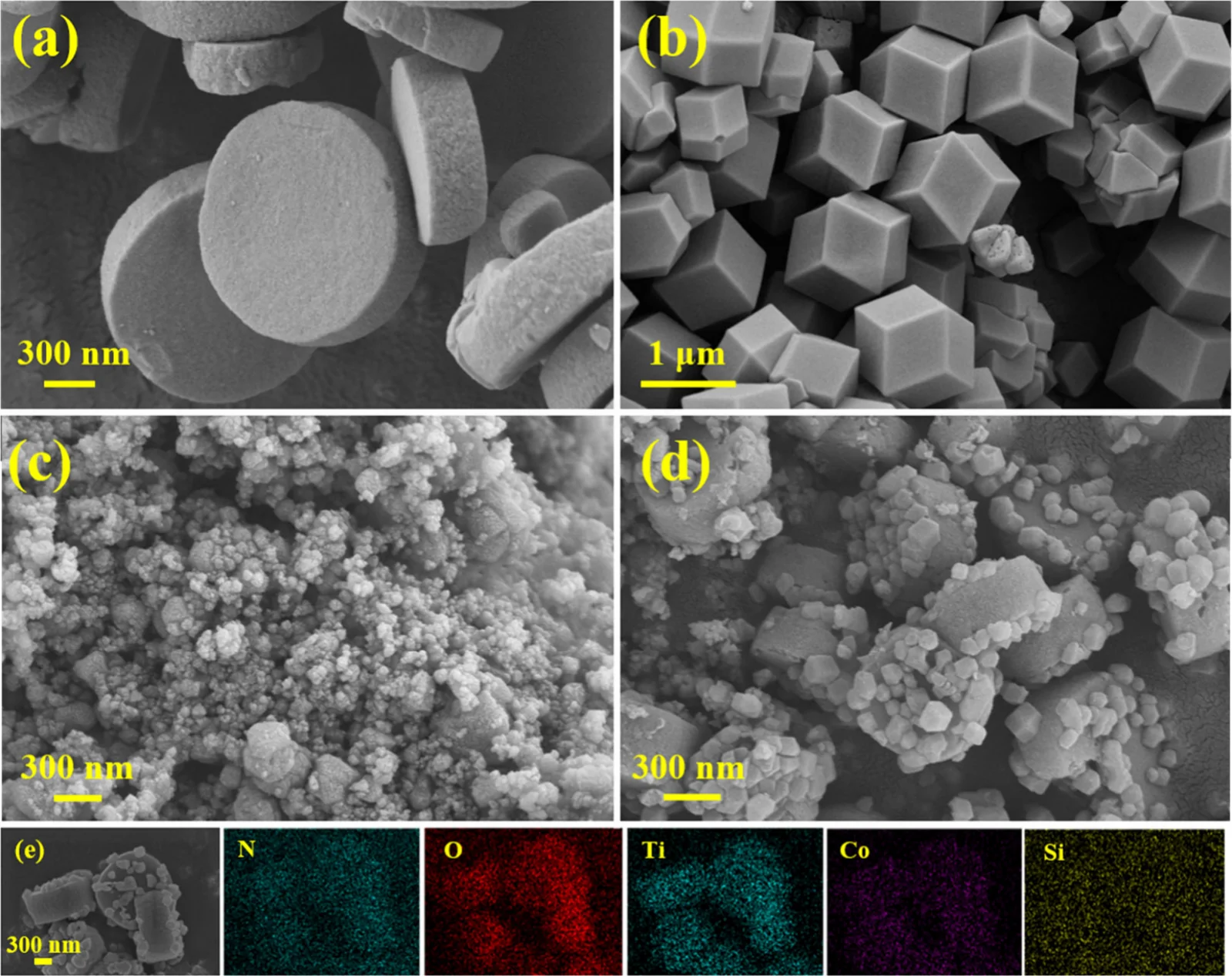
SEM images of (a) MIL-125, (b) ZIF-67, (c) SiC, (d) MIL@ZIF-67/SiC 1:2, (e) SEM-EDS images of MIL@ZIF-67/SiC 1:2.

The SEM images of MIL@ZIF-67/SiC composites with different weight ratios (1:1, 1:2, 1:3) were shown in Figure 2d and Figure S1. The ZIF-67 particles formed on the MIL-125 surface retain their characteristic morphology, indicating that the MOFs-on-MOFs strategy does not affect the sample structure. Moreover, the loaded ZIF-67 particles exhibit significantly smaller sizes compared to pure ZIF-67, suggesting that the presence of MIL-125 may favor the formation of relatively smaller ZIF-67 particles and limit excessive particle growth. This effectively restricts excessive crystal growth of ZIF-67, thereby exposing more active sites. As shown in the figure, the coverage of ZIF-67 on the MIL-125 surface gradually increases with the rising ZIF-67 ratio. At a 1:1 ratio (Figure S1a), ZIF-67 particles are locally distributed on the MIL surface, with portions of the MIL surface remaining smooth. At this point, the ZIF-67 loading is relatively insufficient, potentially limiting the number of available cobalt active sites. At a 1:2 ratio, ZIF-67 particles exhibit relatively uniform distribution across the MIL-125 surface. At the 1:3 ratio (Figure S1b), ZIF-67 densely overlaps and covers the MIL-125 surface, forming a more complete encapsulation structure. This demonstrates that the surface coverage and distribution of ZIF-67 on the MIL surface can be regulated by adjusting the precursor ratio. This structure facilitates efficient contact between reactant molecules and ZIF-67 active sites while preserving the structural support provided by the MIL-125 substrate. Furthermore, the presence of nano-SiC particles between ZIF-67 particles was observed, further confirming the successful synthesis of MIL@ZIF-67/SiC. Additionally, SEM-EDS analysis was performed to investigate the elemental distribution of MIL@ZIF-67/SiC 1:2, as shown in Figure 2e. The elemental mapping confirms the coexistence and spatial distribution of the characteristic elements from the three components, supporting the formation of the composite.

As shown in Figure 3a and Figure S2, the crystal structure of the samples was characterized using XRD. For pure MIL-125, characteristic diffraction peaks were observed at 2θ = 6.72°, 9.7°, and 11.5°, corresponding to typical crystal plane reflections of the MIL-125 framework. The diffraction pattern of ZIF-67 exhibits sharp peaks at 2θ = 7.3°, 10.3°, and 12.7°, consistent with the rhombic dodecahedral crystal structure of ZIF-67 reported in the literature, indicating its excellent crystallinity. For the MIL@ZIF-67/SiC composite material, the characteristic diffraction peaks of MIL-125 remain clearly discernible, while the typical diffraction peaks of ZIF-67 near 7.2° and 12.7° are also evident. Additionally, diffraction signals attributable to SiC are observable near 35.6°. As the ZIF-67 loading ratio increases from 1:1 to 1:3, the relative intensity of ZIF-67 characteristic peaks in the composite material shows a gradual strengthening trend, consistent with the increased ZIF-67 content.

**Figure 3:**
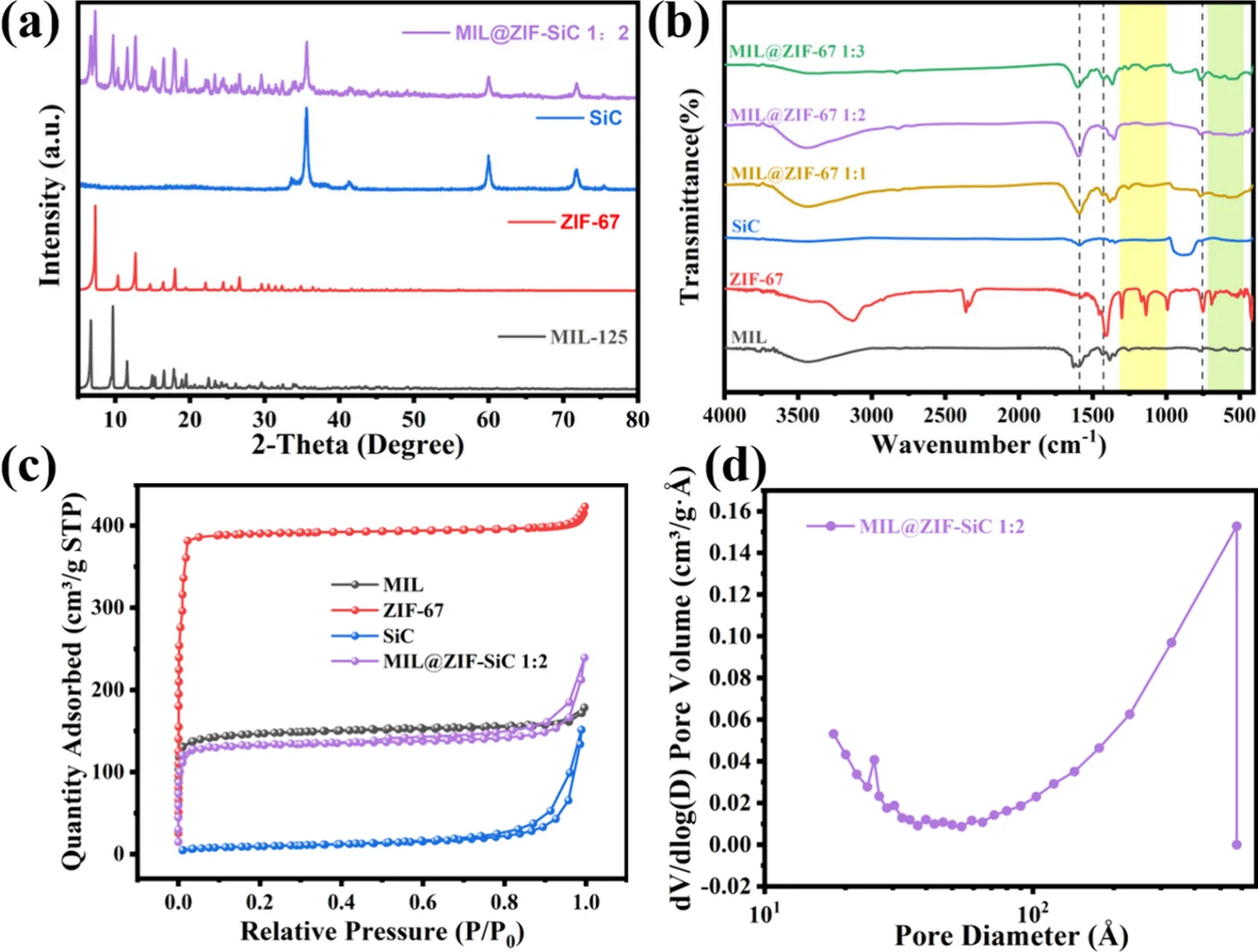
(a) XRD patterns of MIL-125, ZIF-67, SiC and MIL@ZIF-67/SiC 1:2. (b) FT-IR spectra of MIL-125, ZIF-67, SiC, MIL@ZIF-67/SiC, (c) N_2_ adsorption–desorption isotherms of MIL-125, ZIF-67, SiC, and MIL@ZIF-67/SiC 1:2, (d) pore size distribution of MIL@ZIF-67/SiC 1:2.

In the region near 33°, some diffraction peaks of ZIF-67 and MIL-125 are relatively close, with the composite exhibiting a certain degree of broadening or overlap at this point. This may be associated with possible interactions between the two phases. Similarly, the characteristic peak of SiC at 35.6° overlaps with certain diffraction regions of the MOF. At this position, the composite material does not exhibit simple peak superposition but rather peak broadening or intensity changes. This suggests that SiC does not exist as a simple physical mixture but rather exhibits a certain degree of bonding or embedding with the MOF matrix. No significant impurity peaks were observed in the XRD patterns of all composite samples, nor was any significant decrease in crystallinity noted. This indicates that during the incorporation of SiC and the construction of the MOF-on-MOF structure, the crystalline structures of MIL-125 and ZIF-67 were well preserved, with no noticeable phase separation or amorphization occurring. In summary, the XRD analysis results confirm at the crystalline structure level that MIL-125 and ZIF-67 successfully formed a MOF-on-MOF heterostructure, while SiC was also effectively integrated into the composite material system.

Analysis of the FT-IR spectrum data in Figure 3b reveals distinct structural signals from each component across key characteristic vibration regions in the prepared MIL@ZIF-67/SiC composite. For pure MIL-125, the absorption peak observed in the 450–700 cm^–1^ range corresponds to the characteristic vibration of Ti–O clusters, which is associated with the framework vibration of the titanium–oxygen subunits in MIL-125.^47^ Additionally, absorption peaks near 1380 cm^–1^ and 1570 cm^–1^ correspond to the symmetric stretching vibration of the C–O bond in the terephthalate group and the asymmetric stretching vibration of the carboxyl group in the ligand, respectively. The infrared spectrum of ZIF-67 exhibits characteristic absorption features typical of the 2-methylimidazole ligand.^48^ The absorption peak at 755 cm^–1^ is attributed to the out-of-plane bending vibration of the imidazole ring,^49^ while the broad absorption band in the 980–1300 cm^–1^ range corresponds to the breathing vibration of the imidazole ring and the C–N stretching vibration.^50^ The absorption peaks at 1427 cm^–1^ and 1590 cm^–1^ are attributed to the stretching vibration modes of C=N and C=C, respectively.^51^ For SiC samples, a broad and intense absorption band appears in the 800–980 cm^–1^ range, which is attributed to the stretching vibration of the Si–C bond^46^ and is consistent with the characteristic infrared response of silicon carbide materials. For MIL@ZIF-67/SiC composites, the FT-IR exhibits the superposition of characteristic absorption peaks from each individual component. Specifically, vibrational absorption attributable to Ti–O clusters in MIL-125 remains observable in the 450–700 cm ^–1^ range; characteristic absorption of SiC persists within the 800–980 cm^–1^ range; simultaneously, characteristic absorption peaks of ZIF-67 at 755 cm ^–1^, 980–1300 cm ^–1^, 1427 cm ^–1^, and 1590 cm ^–1^ are all present in the composite material. Notably, the intensity of characteristic absorption peaks attributable to ZIF-67 ligands exhibits an increasing trend with rising ZIF-67 loading. Furthermore, no significant peak shifts or new peak formations were observed for any characteristic peaks in the composite material, indicating that the chemical structures of all components are well-preserved during the composite formation process, with all three coexisting within the same system.

Additionally, the specific surface area and pore structure of the samples were analyzed via N_2_ adsorption–desorption isotherms in Figure 3c and Figure S3. Results revealed that pure MIL-125 exhibited typical Type I isotherm characteristics, indicating its abundant micropore structure conducive to pre-enrichment and diffusion of reactant molecules. ZIF-67 similarly demonstrated high nitrogen adsorption capacity, reflecting its favorable micropore properties.^52^ Compared to the single component, the isotherm profile of the MIL@ZIF-67/SiC composite still exhibits Type I characteristics, but the adsorption capacity shows systematic variation with the ZIF-67 loading ratio. Notably, the composite with a 1:2 ratio exhibits the highest nitrogen adsorption capacity at relatively low relative pressures, suggesting a relatively higher microporous surface area and pore volume. This structural foundation provides favorable conditions for the full exposure of active sites and rapid transport of reactants.

The pore size distribution diagrams were shown in Figure 3d and Figure S4. It reveals that both MIL-125 and ZIF-67 exhibit predominantly micropores. The pore size distribution curve of the composite material demonstrates excellent inheritance of the pore characteristics from both components, with no evident signs of pore blockage or structural collapse. The 1:2 sample exhibits a more concentrated pore size distribution with a relatively higher proportion of micropores, indicating that its pore structure was well-preserved and optimized during the composite formation process. This favorable pore structure not only exposes more active cobalt sites but also effectively reduces diffusion resistance, allowing PMS and CIP molecules to reach the active centers more readily, thereby enhancing overall reaction efficiency. Overall, the 1:2 ratio achieves an optimal balance between specific surface area and pore structure, closely correlating with its subsequent outstanding catalytic performance.

### 3.2 Degradation Performance Testing

Figure 4 demonstrates the degradation capacity of different samples toward CIP. Overall, the MIL@ZIF-67/SiC series composites exhibited superior degradation performance compared to single-component materials, preliminarily validating the rationality of the MOFs-on-MOFs strategy combined with SiC reinforcement. Regarding the adsorption phase in Figure S5, all catalysts exhibited low adsorption removal rates for CIP. Among them, MIL@ZIF-67/SiC 1:2 showed slightly higher adsorption than other samples, but overall adsorption contributions remained limited. CIP removal primarily relied on the subsequent PMS activation reaction.

**Figure 4:**
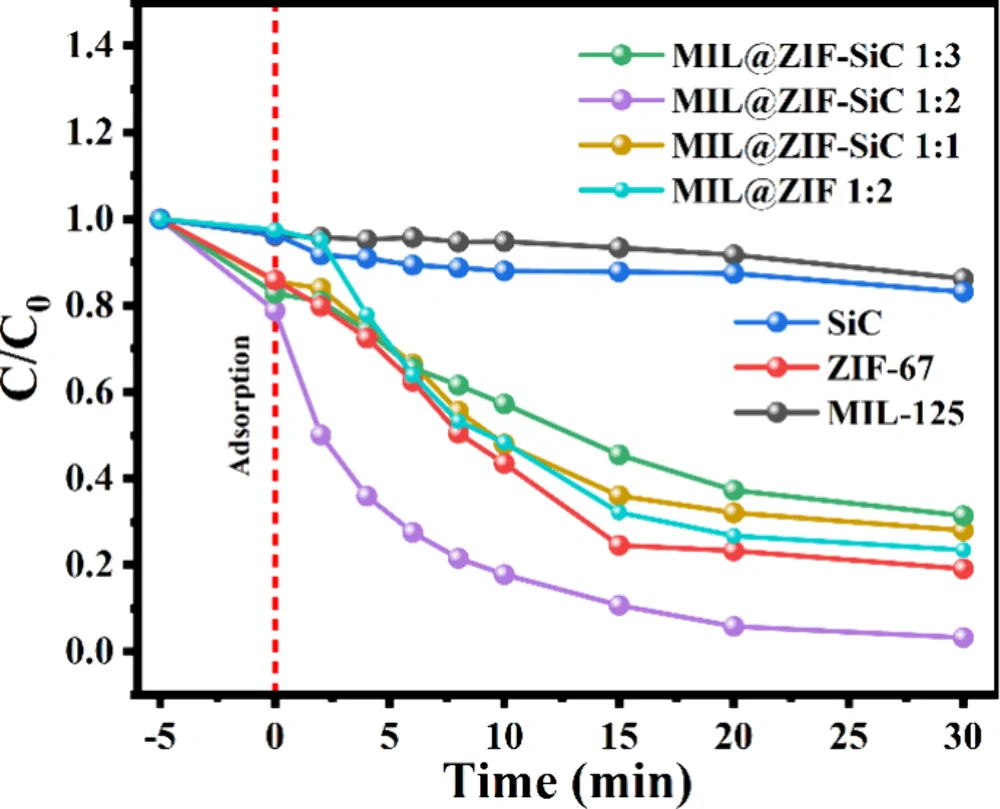
Degradation efficiency of CIP by different catalysts.

As shown in Figure S6, the degradation rate of pure ZIF-67 during the initial reaction phase exceeded that of both MIL@ZIF-67/SiC 1:1 and 1:3 samples. This may be attributed to the fact that at nonoptimal composite ratios, the exposure of ZIF-67 active sites or interfacial mass transfer efficiency was temporarily affected by structural regulation processes, preventing the full realization of synergistic effects. In contrast, MIL@ZIF-67/SiC 1:2 exhibited the fastest kinetics and highest final removal rate throughout the entire reaction, achieving over 96% CIP removal within 30 min. This result indicates that the 1:2 ratio successfully balances active sites, structural stability, and mass transfer efficiency, with overall performance surpassing all single components, including ZIF-67. Furthermore, SiC alone exhibited negligible degradation effect on CIP, indicating its role in the MIL@ZIF-67/SiC system primarily as a carrier and electron mediator rather than a direct participant in PMS activation. The degradation curve of MIL-125 alone showed a relatively slow decline trend, suggesting its limited PMS activation capability. Meanwhile, MIL@ZIF-67 1:2 exhibited inferior degradation performance compared with MIL@ZIF-67/SiC 1:2, indicating that the incorporation of SiC effectively modulates the interfacial electron transfer process between catalytic components, thereby enhancing the catalytic degradation performance. Collectively, these comparisons demonstrate that the MOFs-on-MOFs strategy offers significant advantages in enhancing catalytic performance, while precise control over component ratios plays a critical role in determining final performance outcomes.

To investigate the adaptability of the MIL@ZIF-67/SiC/PMS system in practical applications, this study systematically examined the effects of catalyst dosage, PMS concentration, initial CIP concentration, solution pH, reaction temperature, and different pollutant types on its degradation performance. Catalyst dosage (Figure 5a) is one of the key factors influencing degradation efficiency. When the dosage increased within the range of 10–100 mg/L, the reaction rate constant significantly rose from 0.101 min^–1^ to 3.408 min^–1^ (Figure S7a), accelerating the degradation rate. This is primarily attributed to the increased number of active sites on the catalyst, which promotes the activation of more PMS molecules, thereby generating more active species such as sulfate radicals (SO_4_^•–^). However, when the dosage continued to increase to 100 mg/L, the rate constant growth slowed. This may be due to catalyst particle aggregation at high concentrations, leading to a decrease in effective specific surface area and mass transfer limitations, thereby diminishing the benefits of further increasing the dosage.

**Figure 5:**
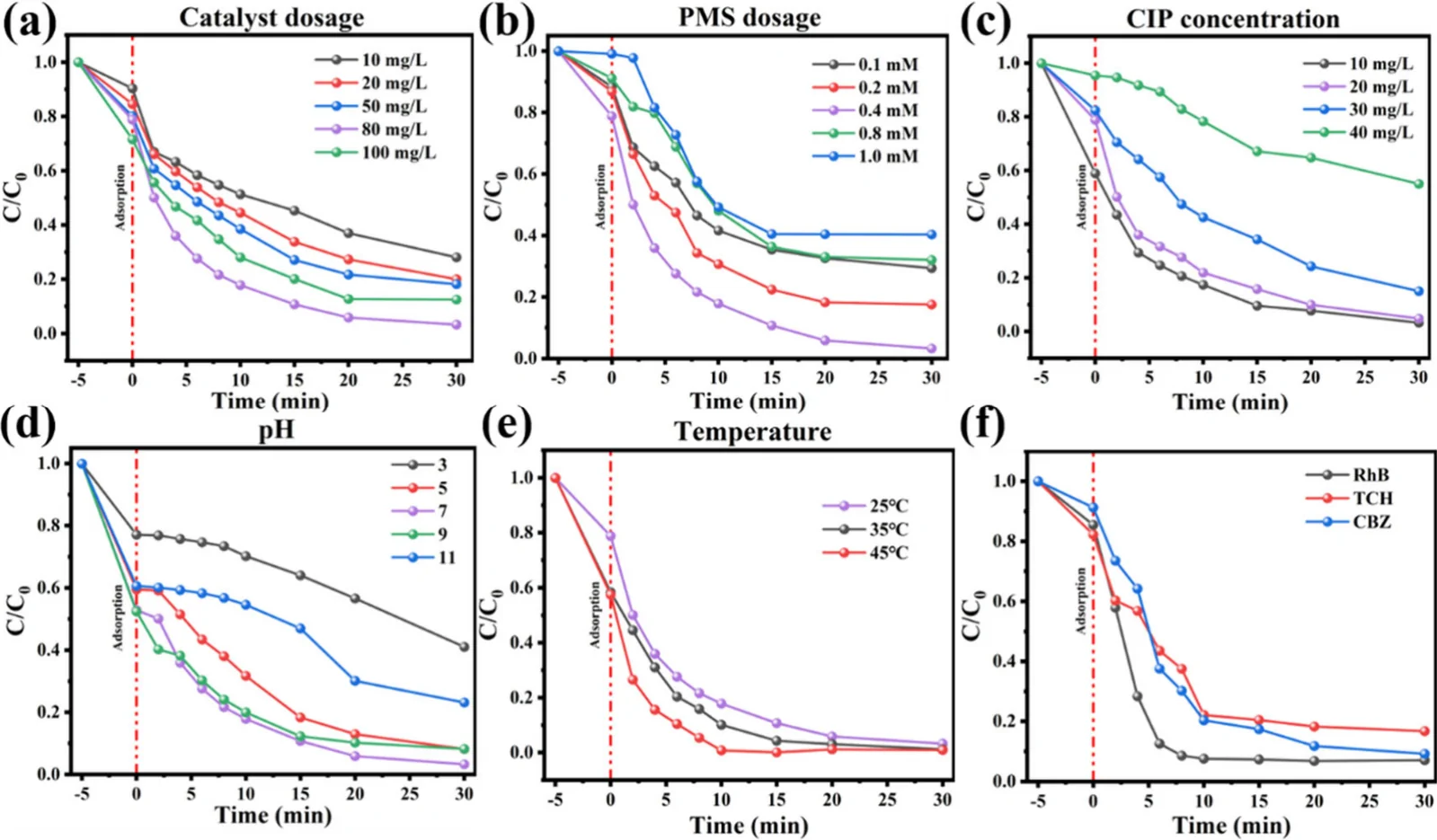
Effects of (a) catalyst dosage, (b) PMS dosage, (c) CIP concentration, and (d) initial pH, (e) temperature on the degradation rate of CIP in the MIL@ZIF-67/SiC 1:2/PMS system. (f) Effect of other contaminants in the MIL@ZIF-67/SiC 1:2/PMS system.

PMS dosage (Figure 5b) showed a positive correlation with degradation rate within the range of 0.4–1.0 mM. When PMS concentration increased from 0.4 mM to 1.0 mM, Kobs rose from 0.238 min ^–1^ to 3.408 min ^–1^ (Figure S7b), indicating that sufficient oxidant supply is essential for maintaining efficient radical chain reactions. Higher PMS concentrations provided more activatable precursors, ensuring continuous generation of reactive oxygen species within the reaction system. However, further increasing PMS concentration paradoxically reduced degradation rates, likely due to excess PMS acting as a radical scavenger. When PMS concentration becomes excessively high, it competes with the target pollutant CIP. Excess PMS captures the generated SO_4_^•–^ in the system, undergoing a side reaction to form the less reactive SO_5_·^–^. This reduces the number of effective radicals available for CIP degradation, consequently lowering the reaction rate. Furthermore, excess PMS may induce mutual quenching among radicals, further diminishing the system’s oxidative capacity. Consequently, an optimal PMS dosage range exists in practical applications, where excessive addition does not enhance degradation efficiency.

3 $$SO_{4}\cdot^{-}+HSO_{5}^{-}\to SO_{5}\cdot^{-}+SO_{4}^{2-}H^{+}$$

As the initial CIP concentration (Figure 5c) increased from 10 mg/L to 40 mg/L, *k*_*obs*_ gradually decreased (Figure S7c), with the degradation rate dropping from 95.2% to 45.0% within 30 min. This indicates that under fixed catalyst and PMS dosage conditions, the total amount of contaminant the system can degrade is limited. High CIP concentrations lead to rapid radical depletion, thereby reducing degradation efficiency.

The initial pH (Figure 5d) ranged from 3.0 to 11.0, with the system exhibiting higher activity under neutral to alkaline conditions (pH = 7 and 11). The highest *k*_*obs*_ (Figure S7d) was observed at pH = 7 (3.410 min ^–1^). The alkaline environment likely promotes deprotonation of the catalyst surface, enhancing adsorption and electron transfer efficiency toward the anionic PMS (HSO_5_^–^), thereby facilitating activation. In contrast, activity significantly decreased under acidic conditions (pH = 3) to 0.890 min ^–1^. This decline likely stems from two factors: first, protonation of the catalyst surface in acidic conditions generates positive charge, inducing electrostatic repulsion with HSO_5_^–^ and hindering PMS diffusion and adsorption onto active sites; Second, lower pH may disrupt the Co­(II)/Co­(III) redox cycle—a process involving proton-coupled electron transfer. Under acidic conditions, the reduction of Co­(III) to Co­(II) is inhibited, thereby weakening the sustained activation capacity of PMS. Additionally, abundant H^+^ ions may react with radicals, affecting the chain transfer process.

The reaction temperature (Figure 5e) ranged from 25 to 45 °C, with the degradation rate increasing with rising temperature, consistent with the Arrhenius law (eq 4). As temperature increased from 15 to 45 °C, Kobs rose from 0.238 min^–1^ to 4.644 min^–1^ (Figure S7e), yielding an apparent activation energy Ea of 48.6 kJ/mol for this catalytic system. This value falls within the activation energy range for Co-based catalysts activating PMS. Temperature elevation effectively enhanced both PMS decomposition and radical generation rates while lowering the reaction’s energy barrier, indicating that this catalytic activation process exhibits characteristics of an endothermic reaction.

4 $$\ln k=\ln A-\frac{E_{a}}{RT}$$

In eq 4, k represents the reaction rate constant in units of concentration, *A* denotes the pre-exponential factor, *E*_*a*_ is commonly referred to as the activation energy, *R* represents the molar gas constant, and *T* denotes the absolute temperature.

Broad-spectrum degradation performance (Figure 5f) testing demonstrates that this system exhibits high-efficiency removal capabilities for multiple organic pollutants. Within 30 min, degradation rates for tetracycline hydrochloride (TCH), Rhodamine B (RhB), and carbamazepine (CBZ) reached 92.9%, 83.2%, and 90.8%, respectively. These three target pollutants represent distinct classes of environmental micropollutants: TCH is a tetracycline antibiotic, RhB is a triphenylmethane dye, and CBZ is a clinically prevalent nitrogen-containing heterocyclic drug. Despite significant structural differences, all three achieved removal rates exceeding 80% after 20 min of reaction within the MIL@ZIF-67/SiC/PMS system. This phenomenon suggests that the active species generated by this catalytic system may possess a broad oxidation spectrum, enabling reactions with organic molecules of varying electron density. From a practical application perspective, the system’s universal removal capability for different pollutant types confers certain advantages when treating complex water qualities.

As shown in Figure S8, in experiments simulating real aquatic environments, the degradation performance of the MIL@ZIF-67/SiC/PMS system toward CIP was significantly affected by coexisting inorganic ions. The experiment examined four common ions (HCO_3_^–^, H_2_PO_4_^–^, SO_4_^2–^, Cl^–^, all at 5 mM concentration), revealing significant differences in their effects on the reaction rate constant (Figure S9). SO_4_^2–^ exhibits the strongest inhibitory effect, likely due to its structural similarity to the activator PMS (HSO_5_^–^). This similarity leads to competitive adsorption at active sites on the catalyst surface (such as Co^2+^), directly hindering PMS access and activation. Additionally, excess SO_4_^2–^ may influence the decomposition equilibrium of PMS via the common-ion effect or interact with reactive sulfur intermediates generated during the reaction, thereby inhibiting radical chain reactions. HCO_3_^–^ exhibits a secondary but still significant inhibitory effect. This aligns with its characteristic as a typical radical quencher, efficiently trapping SO_4_^•–^ and ·OH to form the less reactive carbonate radical (CO_3_·^–^).^53^ Simultaneously, the addition of HCO_3_^–^ significantly increases the solution pH, potentially shifting the system away from the catalyst’s optimal activity pH range, collectively leading to a decrease in degradation efficiency.^54^ The inhibitory effect of Cl^–^ is relatively pronounced. Cl^–^ can react with active free radicals to form less reactive chlorine oxides,^55^ thereby consuming more oxidizing sulfate ions or hydroxyl radicals. Simultaneously, high concentrations of Cl^–^ may adsorb onto the catalyst surface, slightly interfering with the activation process of PMS. The inhibitory effect of H_2_PO_4_^–^ is relatively the weakest in this system. A possible reason is that on the composite surface, H_2_PO_4_^–^ forms relatively stable surface coordination with Ti sites in MIL-125 or Co sites in ZIF-67. This interaction does not completely deactivate the active sites and may even modulate the local electronic environment to some extent, thereby partially offsetting its radical quenching effect.

The cyclic stability of a catalyst is a key indicator for evaluating its practical application potential. To investigate the structural stability and reusability of the MIL@ZIF-67/SiC composite in the PMS activation system, cyclic degradation experiments were conducted on the catalyst. Cyclic degradation experiments (Figure 6a) demonstrate that the MIL@ZIF-67/SiC 1:2 catalyst maintains a degradation efficiency of over 85% for CIP after five consecutive cycles, exhibiting only a slight decline. This decline may stem from the adsorption and accumulation of minor intermediates on the catalyst surface during the reaction or the slight coverage of active sites. Nevertheless, the catalyst overall exhibits excellent catalytic activity and stability.

**Figure 6:**
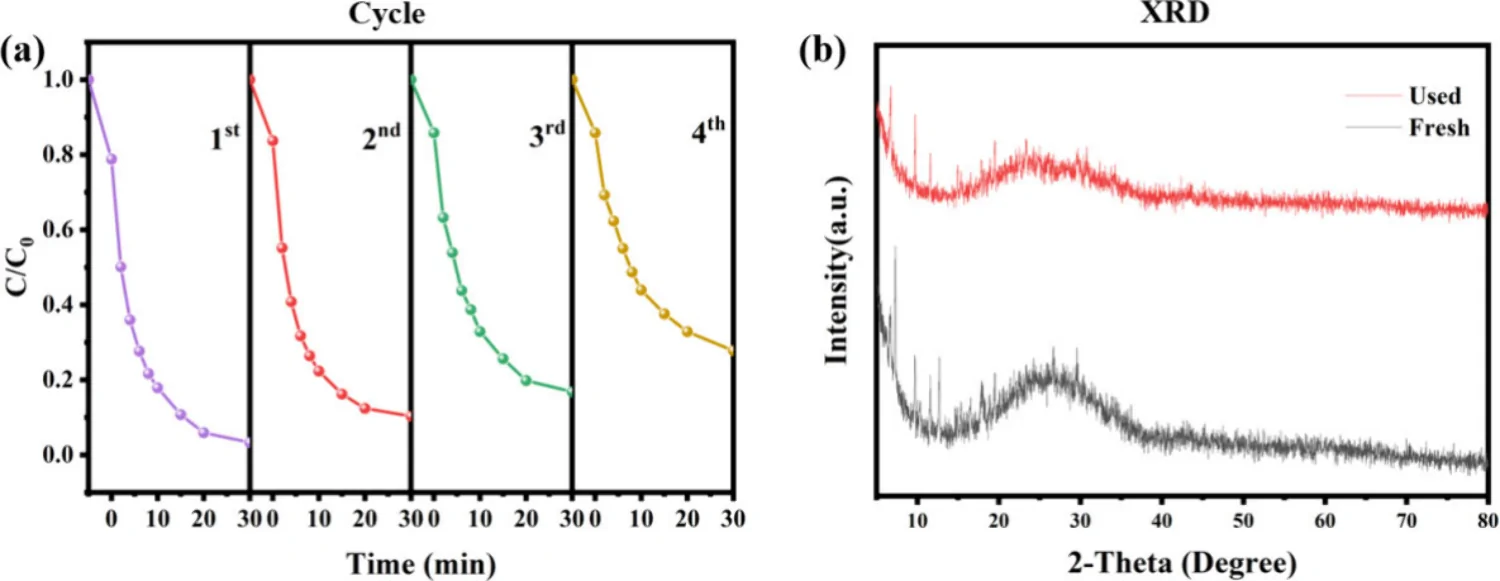
(a) Cyclic stability test of MIL@ZIF-67/SiC 1:2; (b) Comparison of XRD patterns between fresh and used MIL@Z-F-67/SiC 1:2.

Comparing the XRD of the samples before and after the reaction (Figure 6b) reveals that the positions and relative intensities of the main diffraction peaks in the MIL@ZIF-67/SiC composite remained largely unchanged after PMS activation and CIP degradation. Specifically, the low-angle characteristic peaks attributable to MIL-125 remain clearly discernible. The typical diffraction peaks of ZIF-67 near 7.3° and 12.7° are preserved, while the characteristic signal of SiC at 35.6° remains observable. No new diffraction peaks appear in the pattern, and no significant broadening or intensity decay of the original characteristic peaks is observed. These results indicate that the crystalline structures of all components in the composite material maintained good integrity after participating in the PMS activation reaction. The Ti–O framework of MIL-125 did not collapse or undergo amorphization, and the rhombic dodecahedral framework structure of ZIF-67 was maintained. Furthermore, the intensity of diffraction peaks attributable to ZIF-67 in the postreaction sample did not show a significant decrease, indicating that the ZIF-67 particles loaded onto the MIL-125 surface did not undergo noticeable detachment or structural damage. Further SEM observation of the postreaction catalyst morphology (Figure S10) reveals that after multiple cycles, the MIL@ZIF-67/SiC composite retains its original microstructural features. The pancake-like MIL-125 substrate remains clearly visible, uniformly coated with ZIF-67 particles, showing no significant large-area detachment or agglomeration. This observation strongly correlates with the structural stability revealed by XRD analysis, further validating the effectiveness of the MOFs-on-MOFs design in anchoring active sites and preventing active component loss.

### 3.3 Possible Mechanism Analysis

To investigate the primary active species involved in CIP degradation within the MIL@ZIF-67/SiC/PMS system, this study employed radical trapping experiments combined with EPR technology to systematically identify and semiquantitatively analyze reactive oxygen species generated during the reaction process.

In radical scavenging experiments, methanol (MeOH) was selected as a broad-spectrum quencher for SO_4_^•–^ and ·OH, while *tert*-butyl alcohol (TBA) was used for specific quenching of ·OH. As shown in Figure 7a, adding MeOH to the reaction system significantly inhibited CIP degradation, reducing the removal rate from 96% to approximately 40% within 30 min. In contrast, the degradation curve in the TBA-treated system showed only a slight decrease compared to the control, with the removal rate remaining above 90%. This comparison indicates that SO_4_^•–^ plays a dominant role in this catalytic system, while the contribution of ·OH is relatively limited. Both MeOH and SO_4_^•–^ exhibit high reaction rate constants with ·OH, whereas TBA reacts much faster with ·OH than with SO_4_^•–^. Therefore, the addition of TBA did not significantly inhibit degradation, further confirming the dominant role of SO_4_^•–^ in the system.

**Figure 7:**
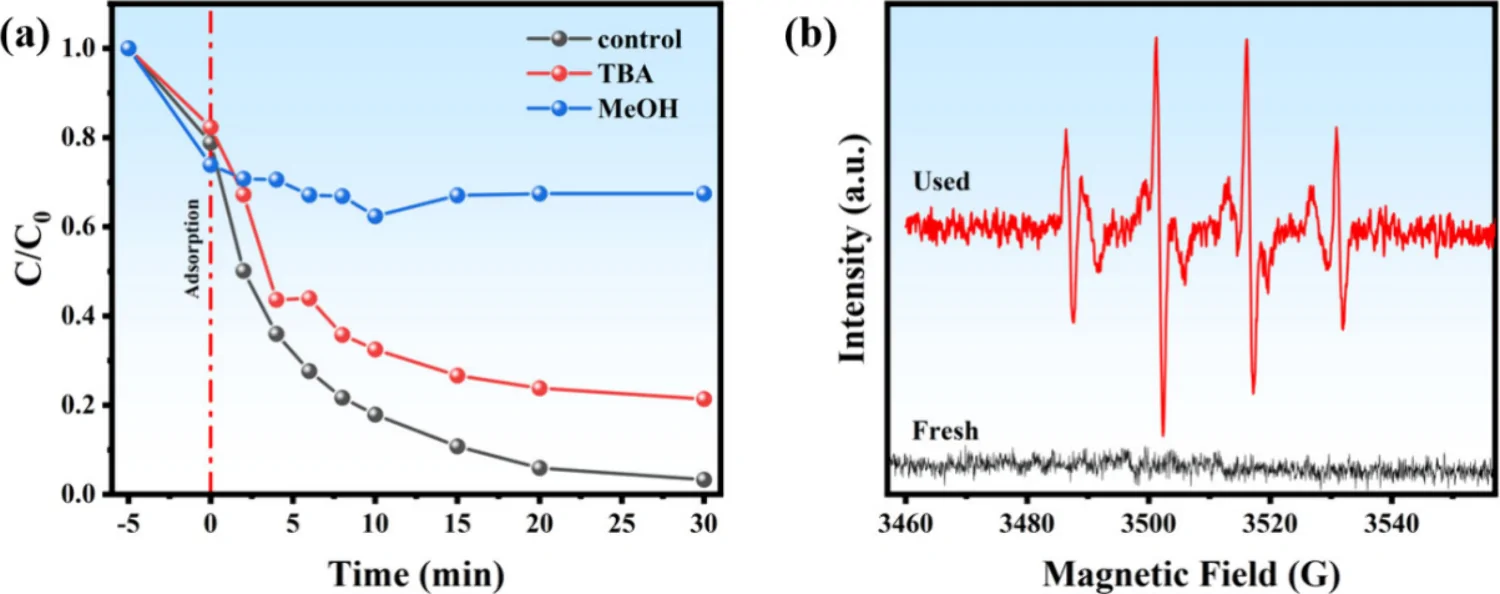
(a) Free radical scavenging experiment and (b) EPR spectra of ·OH/SO_4_^•–^.

To further validate the above conclusions and visually demonstrate radical generation, radical detection in the reaction system was performed using EPR technology combined with the spin trap DMPO (5,5-dimethyl-1-pyrroline-N-oxide). As shown in Figure 7b, no significant EPR signal was detected in the control system containing only PMS, indicating that PMS spontaneously decomposes to generate radicals with difficulty in the absence of a catalyst. However, upon introducing the MIL@ZIF-67/SiC catalyst, a distinct set of characteristic EPR signals was detected immediately after reaction initiation. The peak shapes correspond to the superimposed signals of DMPO-SO_4_^•–^ and DMPO-·OH adducts. Specifically, DMPO-SO_4_^•–^ exhibits a characteristic sextuple peak, while DMPO-·OH displays a typical quadruple peak. Semiquantitative analysis of signal intensities revealed that the peak intensity of DMPO–SO_4_ was significantly higher than that of DMPO-·OH, indicating that SO_4_^•–^ is the dominant radical species in this system. This finding is highly consistent with the results of radical trapping experiments.

As the reaction time extended, the signal intensity of DMPO-SO_4_^•–^ decreased, while that of DMPO-·OH relatively increased. This may stem from secondary reactions of SO_4_^•–^ with water molecules or OH^–^ to form ·OH (eq 5), or from chain reactions accompanying the decomposition of PMS. Furthermore, the addition of MeOH did not completely halt degradation. The remaining approximately 40% degradation activity may stem from contributions by nonradical pathways present in the system (such as singlet oxygen ^1^O_2_ or surface-activated complexes). However, combined with EPR results, it can be confirmed that the radical pathway remains the primary mechanism for CIP degradation in this system.

5 $$SO_{4}\cdot^{-}+H_{2}O\to SO_{4}^{2-}+\cdot OH+H^{+}$$

Based on the combined results of radical trapping experiments and EPR analysis, it can be concluded that in the MIL@ZIF-67/SiC/PMS system, SO_4_^•–^ is the dominant active species responsible for CIP degradation, while ·OH participates as an auxiliary species. The synergistic interaction between these two species collectively achieves highly efficient removal of the pollutant.

It should be noted that, in addition to SO_4_^•–^ and ·OH, O_2_^•–^ and ^1^O_2_ have also been reported to participate in PMS-based oxidation processes involving ZIF-67 and other Co-containing catalysts. However, O_2_^•–^ and ^1^O_2_ were not directly investigated in the present study using selective scavengers or dedicated EPR measurements, and their potential contribution therefore cannot be completely excluded. Nevertheless, the radical-quenching and EPR results obtained in this work provide direct experimental support for the involvement of SO_4_^•–^ and ·OH. The stronger inhibition caused by methanol than by tert-butanol indicates that SO_4_^•–^ makes the dominant contribution to CIP degradation, whereas ·OH plays a comparatively secondary role. Accordingly, the proposed mechanism primarily focuses on a SO_4_^•–^-dominated radical oxidation pathway, while the potential involvement of other ROS is acknowledged.

To investigate the evolution of surface chemical states and the mechanism of active sites in the PMS activation process of the MIL@ZIF-67/SiC catalyst, XPS was employed to characterize the elemental composition and chemical states of the catalyst before and after the reaction (Figure 8). The full-spectrum scan (Figure 8a) reveals characteristic peaks for Co, Ti, O, N, C, and Si in both pre- and postreaction catalysts, indicating the preservation of the catalyst’s main structure after the reaction. However, changes in binding energies and peak shapes within the individual elemental spectra reveal electronic transfers and alterations in the chemical environment occurring on the catalyst surface during the reaction.

**Figure 8:**
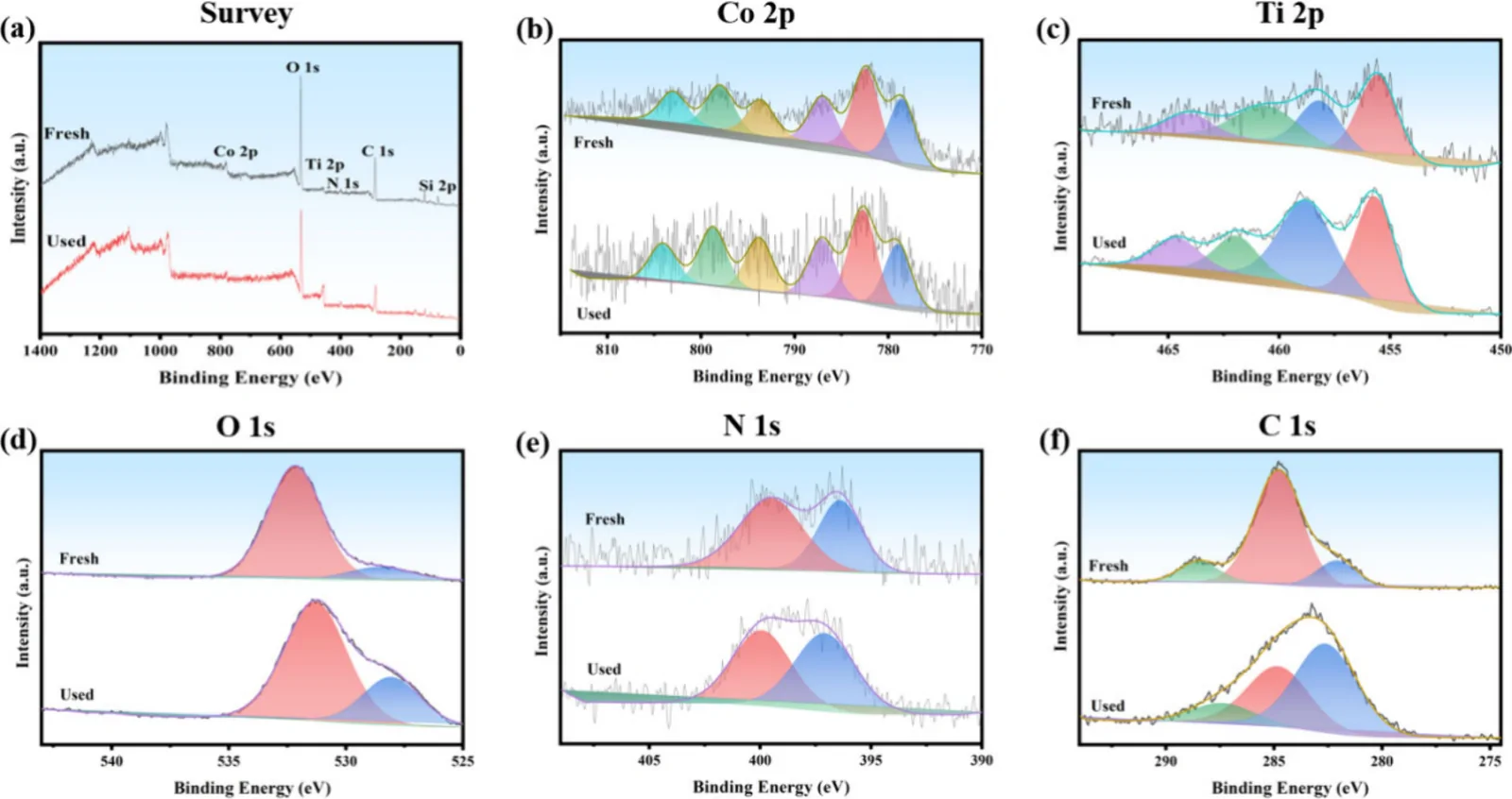
Fresh and postuse XPS spectra of MIL@ZIF-67/SiC 1:2, including (a) full spectrum, (b) Co 2p, (c) Ti 2p, (d) O 1s, (e) N 1s, (f) C 1s.

Figure 8b displays the high-resolution Co 2p spectra of fresh and spent catalysts. In the fresh catalyst, two main peaks at 780.8 and 796.3 eV correspond to Co 2p3/2 and Co 2p1/2, respectively. The accompanying satellite peaks (786 and 803 eV) confirm the presence of Co­(II). After reaction, the binding energy of Co 2p3/2 shifts slightly positive (approximately 0.3 eV), while the satellite peak intensity diminishes slightly. This indicates partial oxidation of Co­(II) to Co­(III). This change aligns closely with the typical mechanism of Co­(II) conversion to Co­(III) during PMS activation. Co­(II) acts as an electron donor to activate PMS, generating SO_4_^•–^, while simultaneously oxidizing itself to Co­(III). Subsequently, Co­(III) can react with PMS or HSO_5_^–^ to regenerate Co­(II), thereby sustaining the catalytic cycle.^53^ In the Ti 2p spectrum of the fresh catalyst (Figure 8c), the two main peaks at 458.5 and 464.2 eV correspond to Ti 2p3/2 and Ti 2p1/2, respectively, reflecting the characteristic oxidation state of Ti­(IV) in MIL-125. Following the reaction, the binding energy of the Ti 2p3/2 peak exhibits a positive shift of approximately 0.2–0.3 eV, while the peak shape remains symmetrical. This indicates a decrease in the electron density surrounding the Ti­(IV) atom, suggesting a degree of electron loss behavior. Between the MIL-125 substrate and the ZIF-67 loading layer, Ti–O clusters and Co nodes form Ti–O–Co bonds via oxygen bridges. When Co­(II) in ZIF-67 serves as the primary electron donor to activate PMS, during electron transfer outward from the Co site, some electrons may be conducted via the Ti–O–Co interface. This temporarily depletes the electron density around Ti, causing Ti­(IV) to exhibit characteristics of electron loss. This indicates that within the MOFs-on-MOFs composite structure, the base metal not only provides structural support but also participates in charge transfer processes through interfacial coupling, thereby synergistically enhancing overall catalytic efficiency.

In the O 1s spectrum (Figure 8d), the spectrum of the fresh catalyst can be fitted into three main components: lattice oxygen (Ti–O) near 529.8 eV, surface hydroxyl or adsorbed oxygen (−OH/adsorbed H_2_O) near 531.5 eV, and adsorbed water or organic oxygen species near 533.0 eV. Following the reaction, the peak intensity at 531.5 eV markedly increased, indicating the formation of additional hydroxyl or adsorbed oxygen species on the catalyst surface. This may stem from intermediates generated during PMS decomposition (such as the adsorption of HSO_5_^–^) or oxygen-containing groups remaining on the surface after radical reactions. It may also be related to altered oxygen affinity resulting from changes in the oxidation state of cobalt. The increase in surface oxygen species promotes interfacial electron transfer, thereby enhancing PMS activation efficiency. In the N 1s spectrum (Figure 8e), two peaks at approximately 398.9 and 400.2 eV correspond to the pyridine-type nitrogen and pyrrole-type nitrogen, respectively, in the 2-methylimidazole ligand of ZIF-67. After the reaction, both the binding energy and relative intensity of these two peaks showed no significant change, indicating that the organic ligands of ZIF-67 maintained chemical stability during the catalytic process without undergoing coordination environment alteration or decomposition. The C 1s spectrum (Figure 8f) can be decomposed into three components: C–C/C=C at 284.8 eV, C–N/C–O at 286.2 eV, and O–C=O at 288.5 eV. The binding energies and proportions of these components remained essentially unchanged after the reaction, further confirming the stability of the organic framework during the reaction process and simultaneously ruling out the possibility of ligand involvement in redox reactions or degradation. Overall, the spectral changes in C, N, and O primarily reflect an increase in surface oxygen species, while the framework structure of the organic ligands remains intact. This is partly attributable to the protective effect of the MIL-125 substrate on the ZIF-67 loading layer, which effectively suppresses the loss of active components.

To identify the potential reactive sites of CIP, density functional theory (DFT) calculations were performed, and the results are shown in Figure 9. The highest occupied molecular orbital (HOMO) possesses a high electron density, making it more susceptible to electrophilic attack by reactive species. As illustrated, the HOMO of CIP is mainly distributed over the piperazine ring and the adjacent quinolone skeleton, indicating that these regions are more vulnerable to electrophilic attack. And the lowest unoccupied molecular orbital (LUMO) is primarily localized around the carbonyl and carboxyl groups on the quinolone ring. As the lowest unoccupied molecular orbital, the LUMO is more prone to nucleophilic attack. The Fukui function further confirms the distribution of reaction sites, where *f* ^–^ represents the electrophilic attack index, *f* ^+^ represents the nucleophilic attack index, and *f* ^0^ represents the radical attack index. The *f* ^–^ values which describe the tendency toward electrophilic attack, are highest for 1­(O) and 15­(N), indicating that the oxygen atoms in the carbonyl/carboxyl groups and the nitrogen atom in the piperazine ring are vulnerable to electrophilic attacks. The *f* ^+^ values representing susceptibility to nucleophilic attack, are mainly concentrated at 4­(C), 22­(O), 23­(O). Moreover, the *f* ^0^ values represent the sites that are more susceptible to radical attack. In the CIP molecule, the sites prone to radical attack are mainly located at O1, N15, O23.

**Figure 9:**
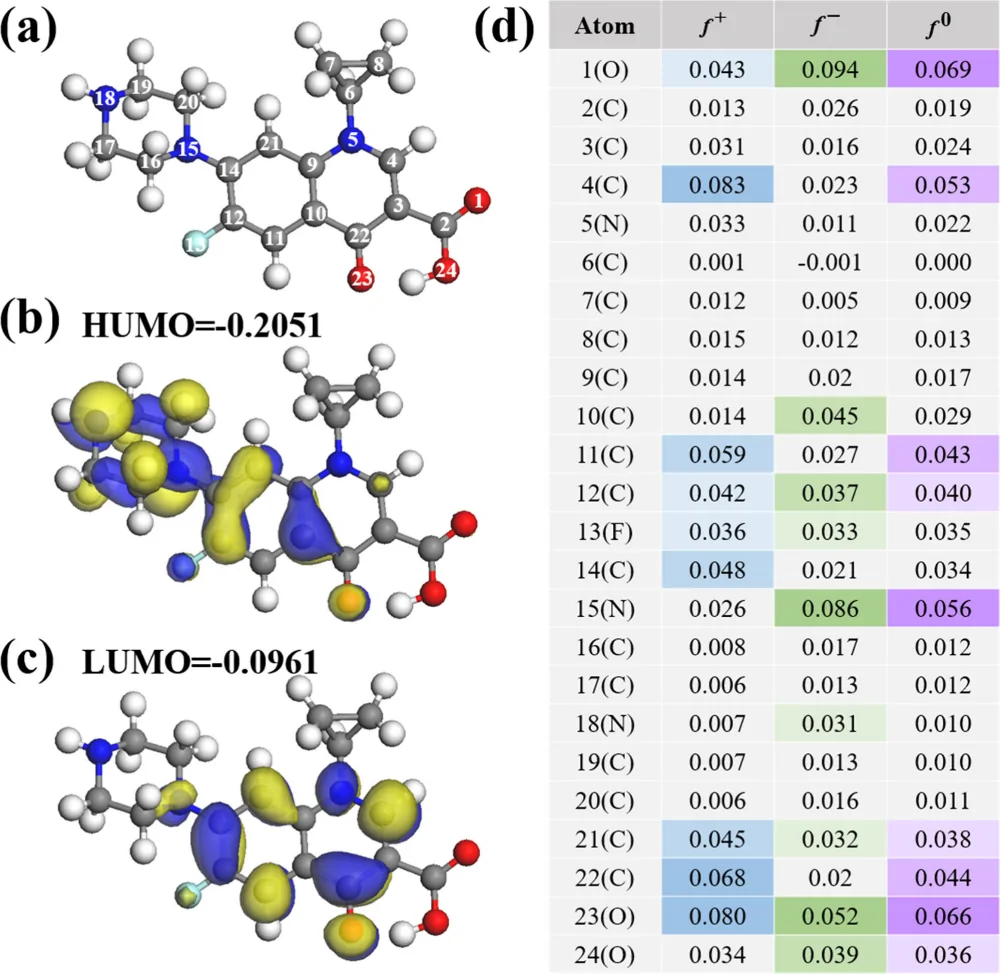
(a) The optimized molecular structure of CIP, (b) HUMO, and (c) LUMO of CIP. (d) Fukui function values of CIP.

In summary, the reaction mechanism of sample-activated PMS degrading CIP is illustrated in Figure 10. Within this composite system, ZIF-67 serves as the primary active component, with its highly dispersed Co^2+^ sites constituting the core for PMS activation. Co^2+^ activates PMS (HSO_5_^–^) via single-electron transfer, generating the highly oxidative SO_4_^•–^ alongside hydroxyl radicals (·OH), while simultaneously oxidizing itself to Co^3+^.

6 $$Co^{2+}+HSO_{5}^{-}\to Co^{3+}+SO_{4}\cdot^{-}+OH^{-}$$

7 $$Co^{2+}+HSO_{5}^{-}\to Co^{3+}+SO_{4}^{2-}+\cdot OH$$

**Figure 10:**
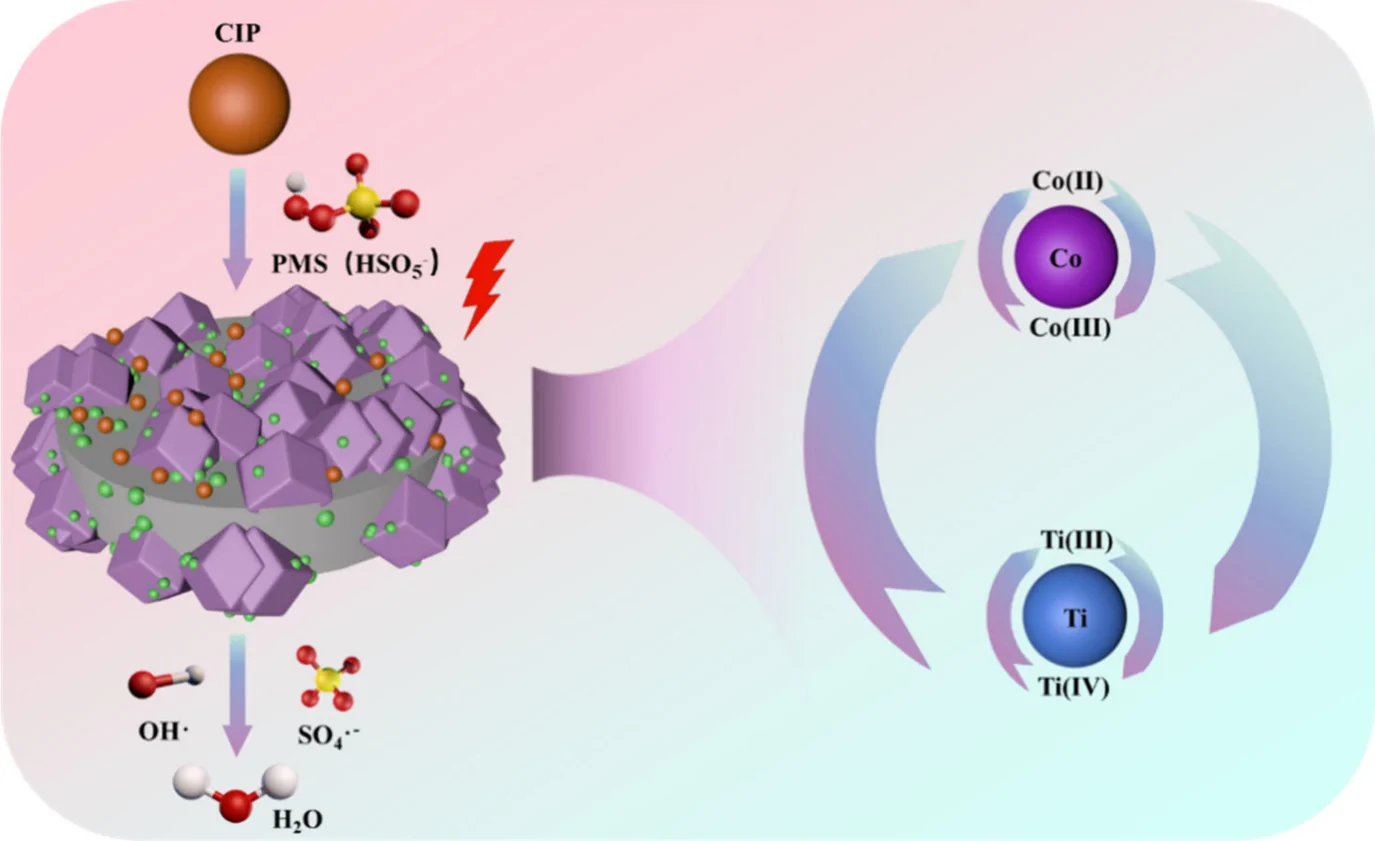
Schematic diagram of CIP degradation mechanism.

XPS analysis results indicate that the binding energy of the Co 2p peak shifts positively postreaction, accompanied by a decrease in satellite peak intensity, confirming the conversion of Co^2+^ to Co^3+^. Concurrently, the binding energy of the Ti 2p peak also exhibits a slight positive shift, suggesting a reduction in electron density around the Ti­(IV) atom. This implies that the Ti–O–Co interface may have participated in the electron transfer process.^56,57^ This phenomenon indicates that the MIL-125 framework not only provides structural support for ZIF-67, but its titanium–oxygen clusters may also facilitate charge migration and redistribution through interfacial coupling,^58^ thereby indirectly promoting the regeneration and recycling of Co sites. During continuous operation of the catalytic cycle, Co^3+^ must be reduced to Co^2+^ to maintain the regeneration of active sites (eq 8). The retention of the Co^2+^ characteristic signal in XPS and the change in Ti oxidation state suggest that this reduction process may rely on interfacial synergistic effects: On one hand, Ti–O clusters within MIL-125 act as electron transfer mediators, facilitating electron migration from electron-rich species in the system to Co^3+^. On the other hand, SiC, serving as a conductive carrier, accelerates interfacial charge redistribution through its exceptional electron transport capability, thereby enhancing the reduction kinetics of Co^3+^ and enabling sustained Co^2+^/ Co^3+^ cycling.

8 $$Co^{3+}+HSO_{5}^{-}\to Co^{2+}+SO_{5}\cdot^{-}+H^{+}$$

SO_4_^•–^, as the dominant radical in the system, can directly attack unsaturated bonds or aromatic rings within the CIP molecular structure, thereby achieving rapid degradation of the pollutant (eq 9). SO_4_^•–^ primarily reacts with electron-rich groups in CIP molecules via electron transfer, leading to C–F bond cleavage, quinolone ring opening, or piperazine ring cleavage, generating a series of intermediate products. Concurrently, although ·OH radicals generated in the system contribute relatively less, they synergistically participate in the degradation process. Possessing strong electrophilicity, ·OH primarily attacks unsaturated double bonds (e.g., C = C, C = O) and aromatic rings in CIP molecules via addition reactions, inducing hydroxylation reactions that promote further transformation and mineralization of pollutants (eq 10). The combined action of both radical species progressively disrupts the CIP molecular structure, ultimately converting it into small-molecule organic acids, CO_2_, and H_2_O.

In summary, the MIL@ZIF-67/SiC composite material utilizes the Co sites in ZIF-67 as the core, where the redox cycle of Co^2+^/ Co^3+^ drives PMS activation to generate a radical pathway dominated by SO_4_^•^. MIL-125 stabilizes the active sites through its Ti–O–Co interfacial structure and participates in charge regulation. SiC serves as an electron conduction medium to enhance interfacial charge migration efficiency. The synergistic interaction of these three components collectively enhances catalytic activity and maintains reaction stability, ultimately achieving efficient degradation of CIP.

9 $$SO_{4}\cdot^{-}+CIP\to \mathrm{int}ermediate\to CO_{2}+H_{2}O+SO_{4}^{2-}$$

10 $$\cdot OH+CIP\to \mathrm{int}ermediate\to CO_{2}+H_{2}O$$

## 4 Conclusion

This study successfully constructed MIL@ZIF-67/SiC composite materials for activating PMS to degrade CIP in water based on the MOFs-on-MOFs strategy. MIL-125 served as the structural substrate, while ZIF-67 was distributed on its surface as the main Co-containing active component, and SiC was introduced as a functional enhancement carrier. This design fully leverages MIL-125’s high specific surface area and structural stability provide a favorable surface for the formation and dispersion of ZIF-67, effectively suppressing cobalt active site agglomeration. SiC, with its excellent electron conductivity, promotes interfacial charge transfer and forms a synergistic enhancement effect with the MOF components. Performance testing indicates that the MIL@ZIF-67/SiC catalyst with an optimized 1:2 ratio exhibits optimal catalytic activity, achieving over 96% CIP degradation within 30 min. It also demonstrates broad-spectrum degradation capabilities for various pollutants including tetracycline, Rhodamine B, and carbamazepine. Mechanism studies reveal that CIP degradation in this system primarily relies on a SO_4_^•–^-dominated radical pathway: Co^2+^ in ZIF-67 serves as the PMS activation core site, generating SO_4_^•–^ via single-electron transfer and oxidizing to Co^3+^; MIL-125 participates in charge regulation via Ti–O–Co interface bonding, with postreaction Ti 2p binding energy shifts indicating the mediating role of titanium oxide clusters in electron transfer; SiC accelerates the reduction of Co^3+^ back to Co^2+^, sustaining continuous catalytic cycling. The composite material exhibits stable crystal structure and chemical composition postreaction, demonstrating excellent structural stability and resistance to leaching. In summary, this study successfully constructed a composite material with both high activity and high stability through the MOFs-on-MOFs strategy and synergistic functional carrier design. This provides a feasible material design solution to address the challenge of balancing activity and stability in advanced oxidation applications using single MOF materials. It also offers theoretical basis and experimental support for expanding the application of functionalized MOF composites in water treatment.

## Supplementary Material



## Notes

Chemicals and reagents, characterization, details of the calculation, additional characterization and degradation reactions (PDF)
