# Supplementary material for: HMIL@ZIF-67/SiC Composite with MOFs-on-MOFs Structure for Activated Persulfate Degradation of Ciprofloxacin
Source: ACS Omega. 2026 Oct 6;11(41):62824–37. doi: 10.1021/acsomega.6c04084 (PMC13688485; doi:10.1021/acsomega.6c04084)
Supplement: Supplementary file 1 [file ao6c04084_si_001.pdf]

Supporting Information

MIL@ZIF-67/SiC Composite with MOFs-on-MOFs Structure for Activated Persulfate Degradation of Ciprofloxacin

Haonan Pei[a]†, Siyuan Liu[a,b]†, Jiaqi Yuan[a], Nannan Wang[a], Yongxin Lei [a]*, Qun Wei [a]*, Yanqiu Zhu[a,b]*

[a] State Key Laboratory of Featured Metal Materials and Life-cycle Safety for Composite Structures, MOE Key Laboratory of New Processing Technology for Nonferrous Metals and Materials, School of Resources, Environment and Materials, Guangxi University, Nanning 530004, China.

[b] Faculty of Environment, Science and Economy, University of Exeter, EX4 4QF, UK.

**Corresponding author: E-mail address: Y.Zhu@exeter.ac.uk (Prof. Yanqiu Zhu)*

† These authors contributed equally to this work.

Text S1 Chemicals and reagents

Tetraisopropyl titanate (TTIP), 2-amino-1,3-benzenedicarboxylic acid (NH_2-BDC), 2-methylimidazole (2-MeIM), cobalt nitrate hexahydrate ($Co(NO_3)_2 \cdot 6H_2O$), polyvinylpyrrolidone (PVP), N, N-Dimethylformamide (DMF), Anhydrous Methanol (MeOH), tert-butanol (TBA), sodium hydroxide (NaOH), sodium hydrogen carbonate ($NaHCO_3$), sodium dihydrogen phosphate (NaH_2PO_4), sodium sulfate (Na_2SO_4), and sodium chloride (NaCl) were purchased from Sinopharm Chemical Reagent Co., Ltd.

Model contaminants: Ciprofloxacin (CIP), Tetracycline hydrochloride (TCH), Rhodamine B (RhB), 5,5'-Dimethyl-1-pyrrolidone-N-oxide (DMPO), and potassium monopersulfate (PMS, $KHSO_5 \cdot 0.5\ KHSO_4 \cdot 0.5\ K_2SO_4$) were purchased from Shanghai McLean Biochemical Technology Co., Ltd.

Text S2 Characterization

The surface morphology of the catalyst was investigated using field emission scanning electron microscopy (FESEM, Carl Zeiss Sigma 300, Germany). The composition of the catalyst was analyzed by X-ray diffraction (XRD, Rigaku D/MAX 2500V, Japan) employing Cu Kα radiation (100 mA, 40 kV) at a scanning rate of 5°/min. The chemical structure and surface functional group types of the material were analyzed using a Fourier transform infrared spectrometer (FT-IR, Thermo Fisher Nicolet IS-50, USA). Nitrogen adsorption-desorption isotherms were measured at -196°C using a multi-station fully automated specific surface area analyzer (Micromeritics ASAP 2020, USA). All samples were degassed under vacuum at 200°C for 12 hours prior to testing. Pore sizes were calculated using the Barrett-Joyner-Haland (BJH) model. Free radicals in the system were detected using electron paramagnetic resonance (EPR, Bruker A300, Germany) with the following parameters: modulation amplitude 3G, microwave power 20.00 mW, microwave frequency 9.79 GHz, modulation frequency 100 kHz. Elemental composition and valence states of the materials were analyzed using X-ray photoelectron spectroscopy (XPS, Axis Ultra DLD, UK) with Al Kα radiation as the source.

To accurately analyze the reaction active sites and establish the reaction pathway, this study conducted computational research using density functional theory. Firstly, a molecular model was constructed using ChemDraw. After geometric optimization using Materials Studio 2023 software, the highest occupied molecular orbital (HOMO), the lowest unoccupied molecular orbital (LUMO), and the electrostatic potential surface of the CIP molecule were calculated at the B3LYP/6-31+G(d,p) basis set level. At the same time, Hirshfeld charge distribution parameters f^-, f^+ and f^0 were calculated and plotted. These calculations provided the electrophilic attack index (f^-), nucleophilic attack index (f^+) and radical attack index (f^0).

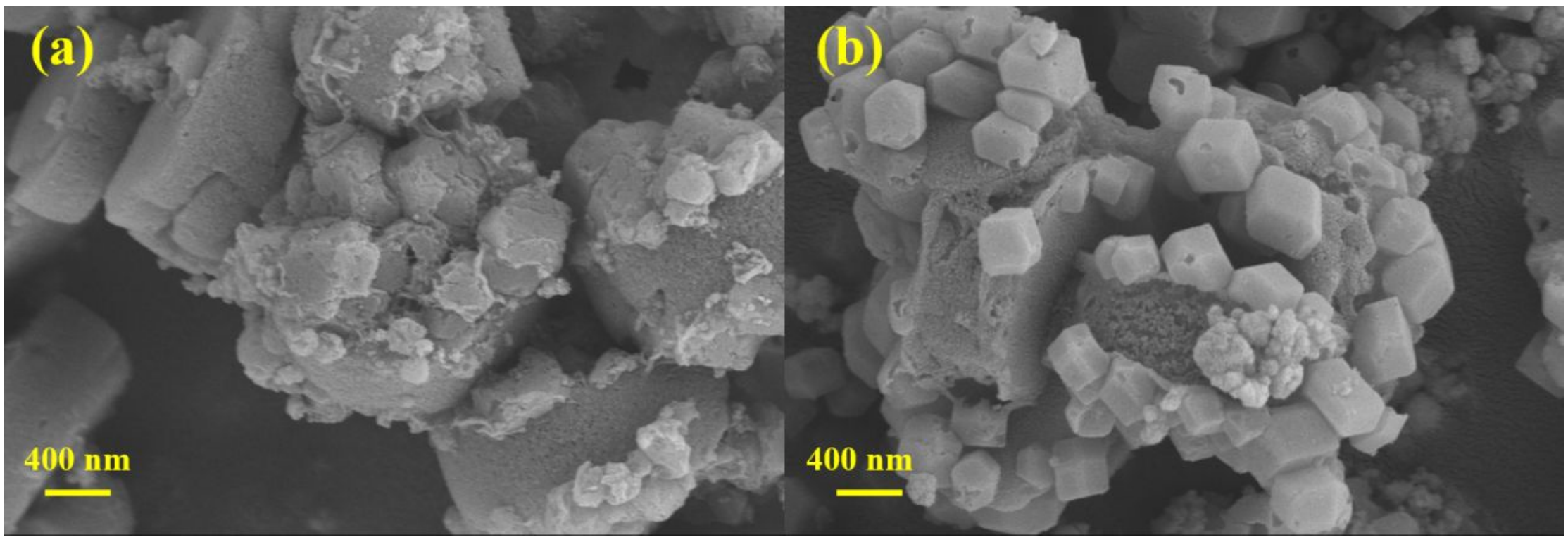

Fig. S1 SEM images of (a)MIL@ZIF-67/SiC 1:1 and (b) MIL@ZIF-67/SiC 1:3.

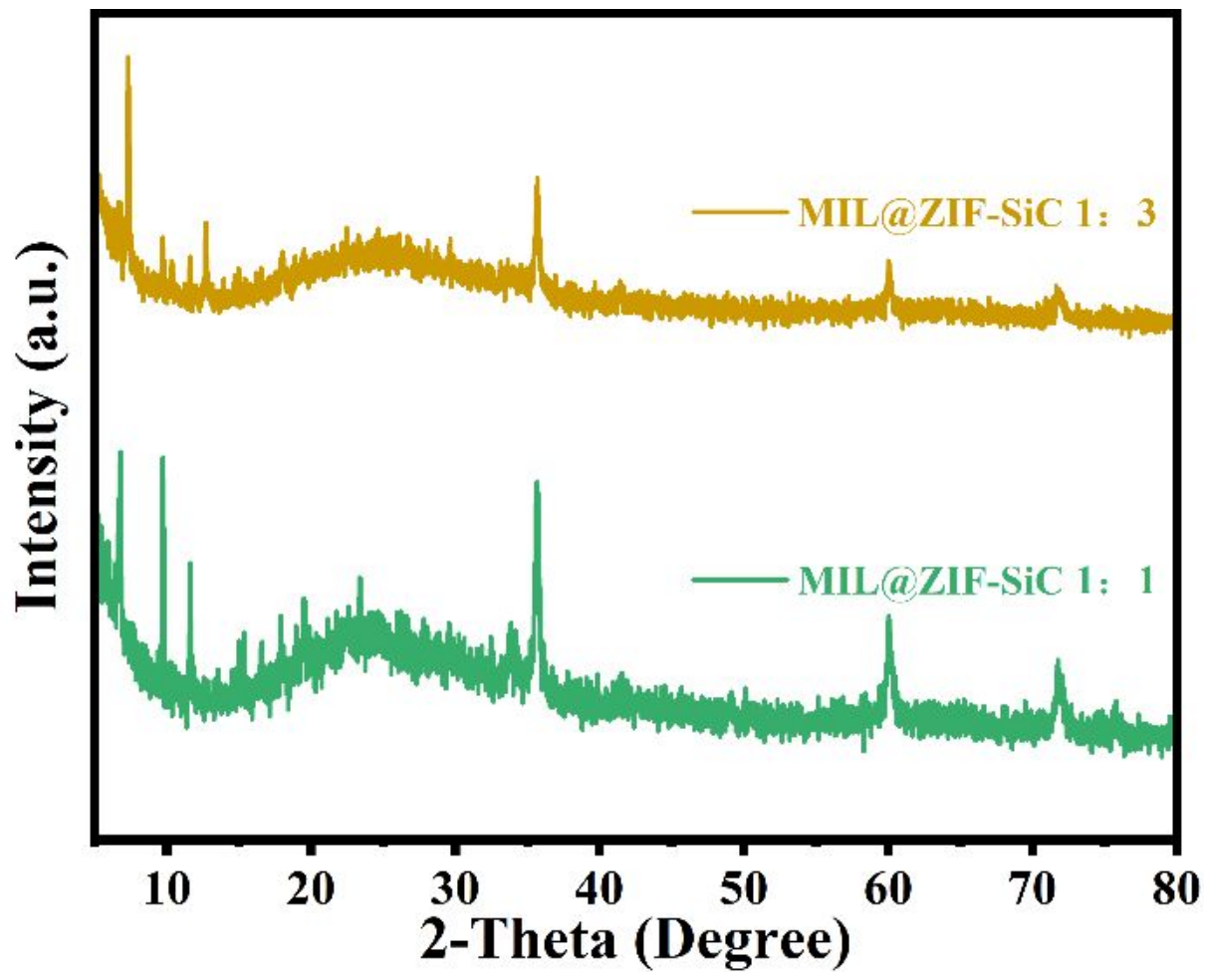

Fig. S2 XRD of MIL@ZIF-67/SiC 1:1 and MIL@ZIF-67/SiC 1:3.

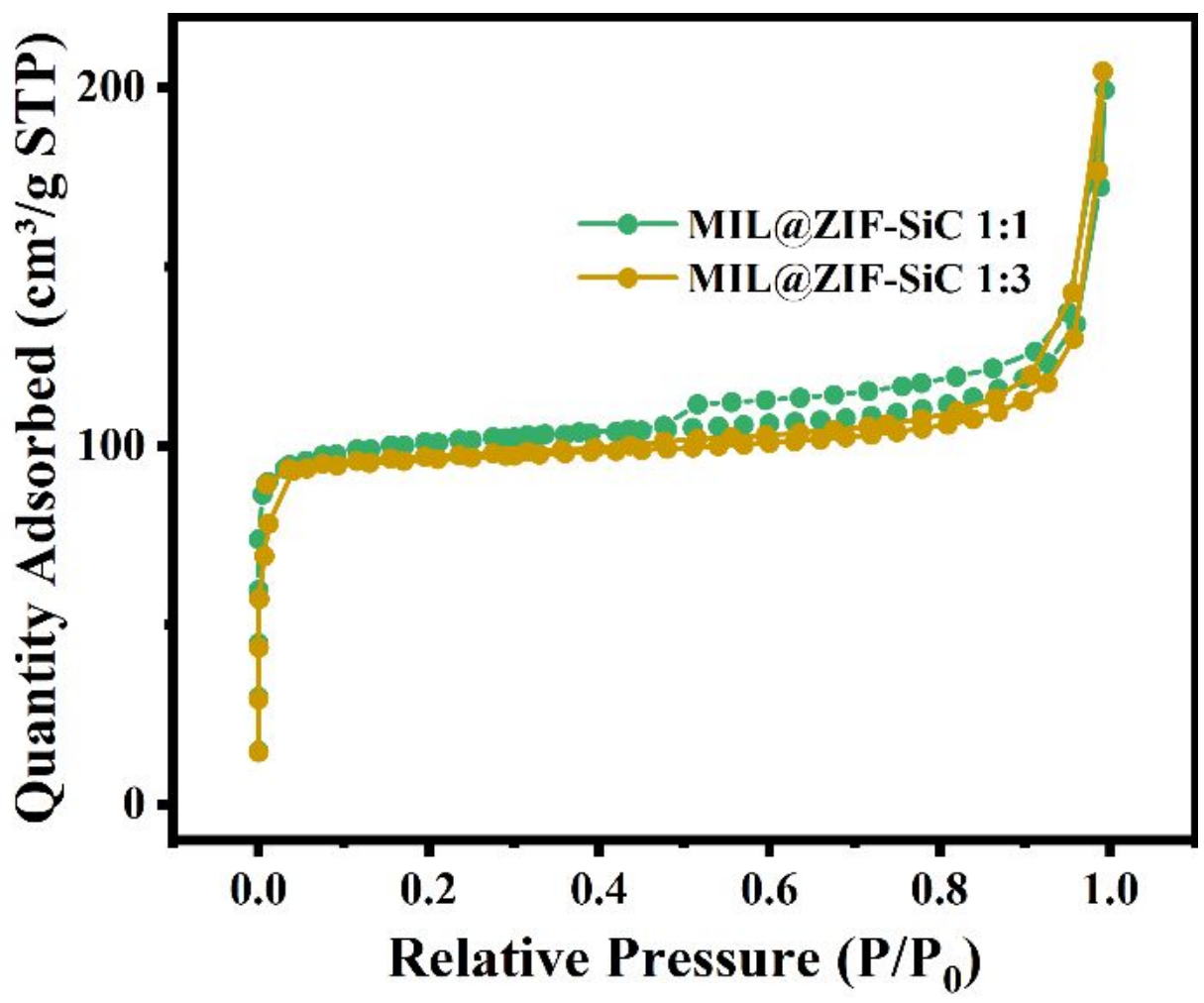

Fig. S3 N_2 adsorption-desorption isotherms of of MIL@ZIF-67/SiC 1:1 and MIL@ZIF-67/SiC 1:3.

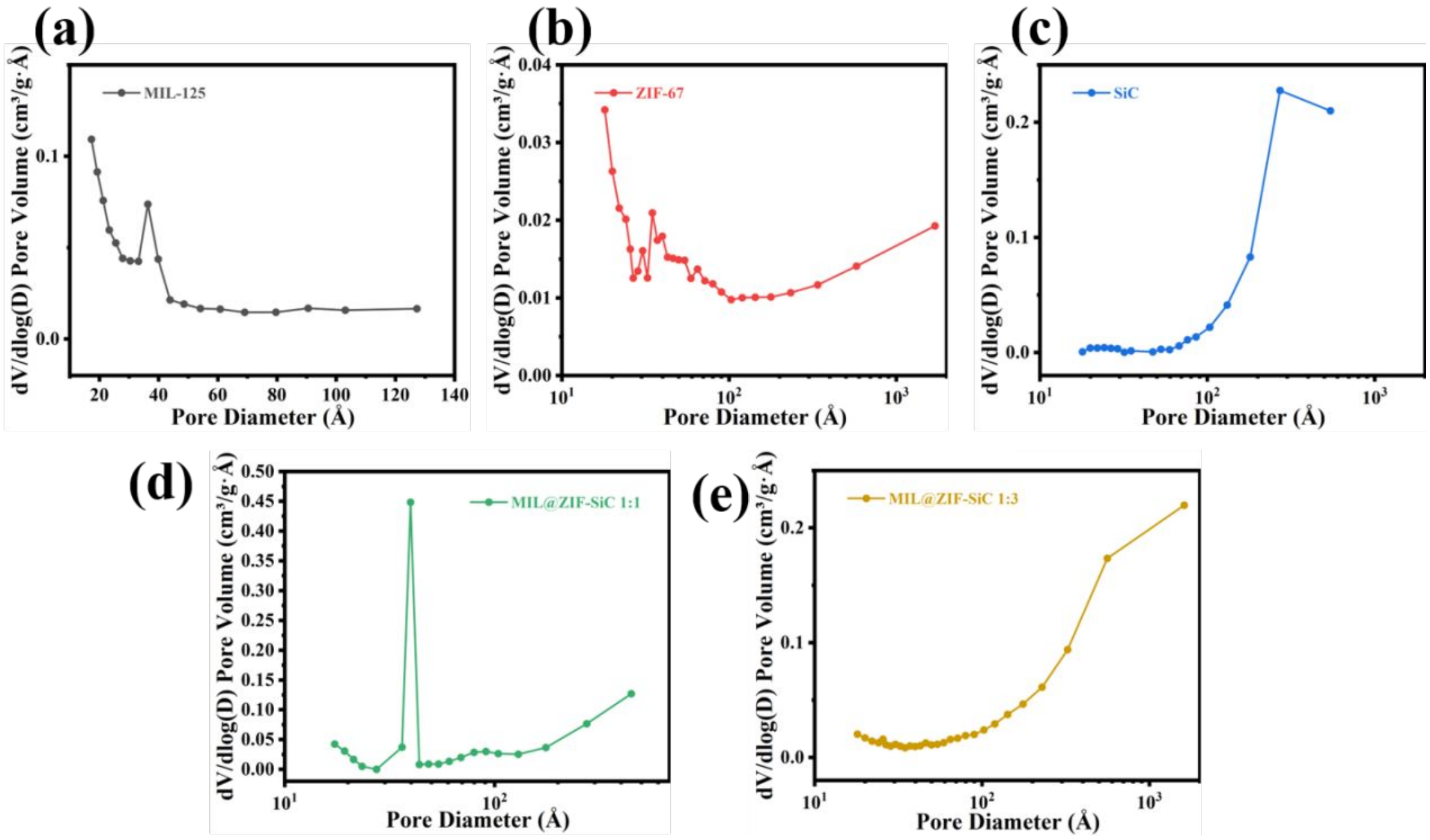

Fig. S4 Pore size distribution of (a) MIL-125, (b) ZIF-67, (c) SiC, (d) MIL@ZIF-67/SiC 1:1, and (e) MIL@ZIF-67/SiC 1:3.

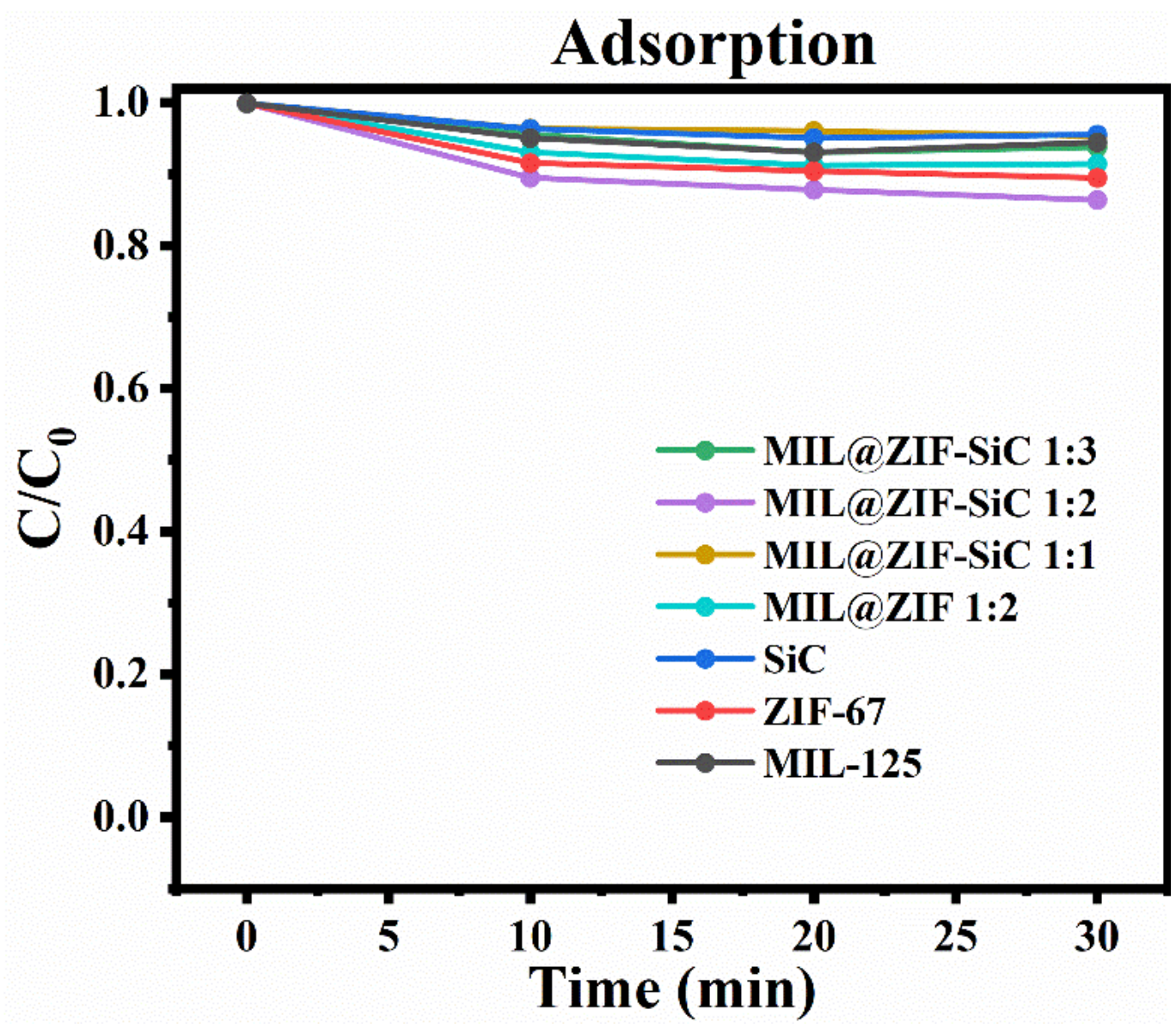

Fig. S5 The adsorption capabilities of different catalysts for CIP.

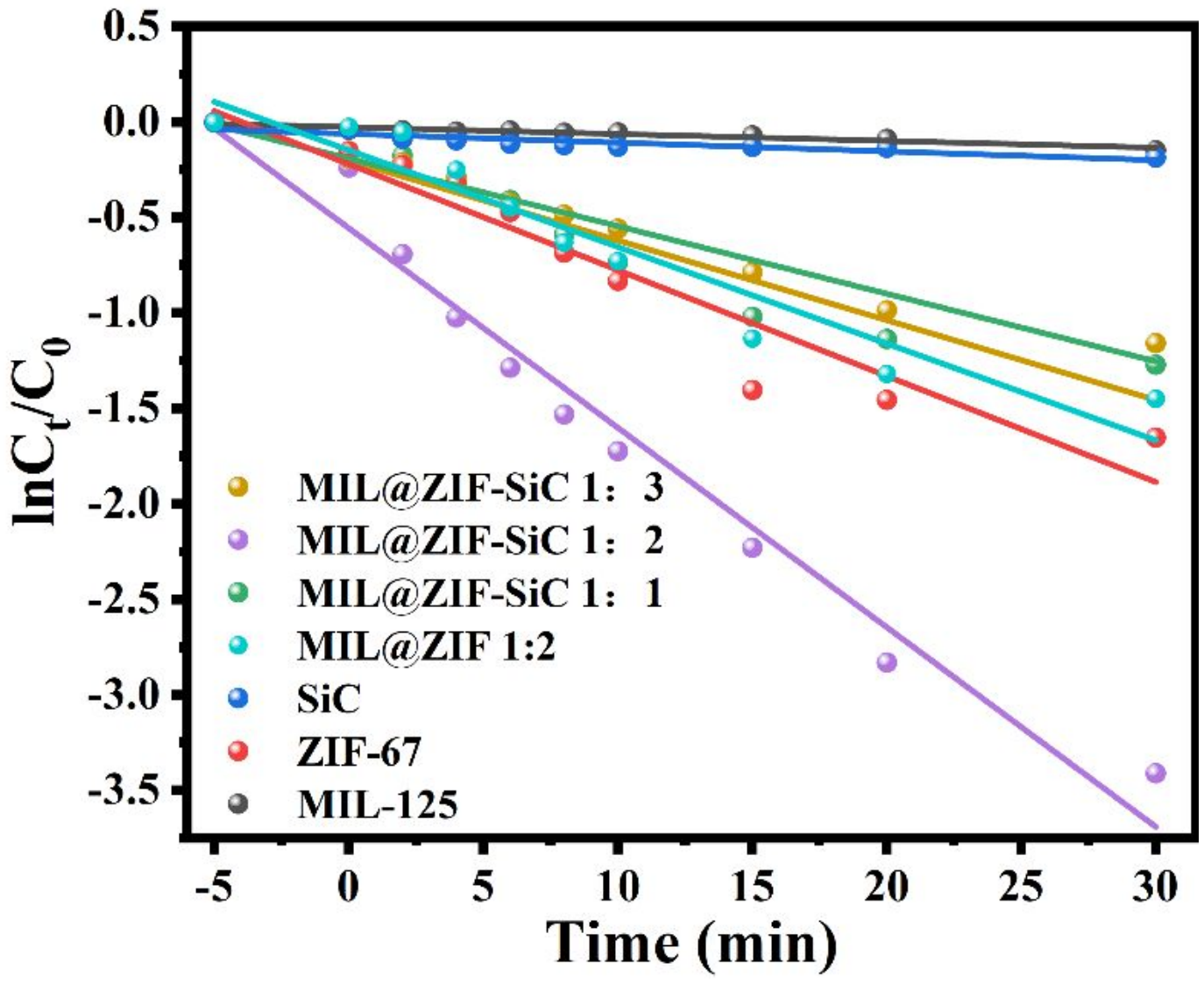

Fig. S6 The corresponding reaction rate constants of different catalysts.

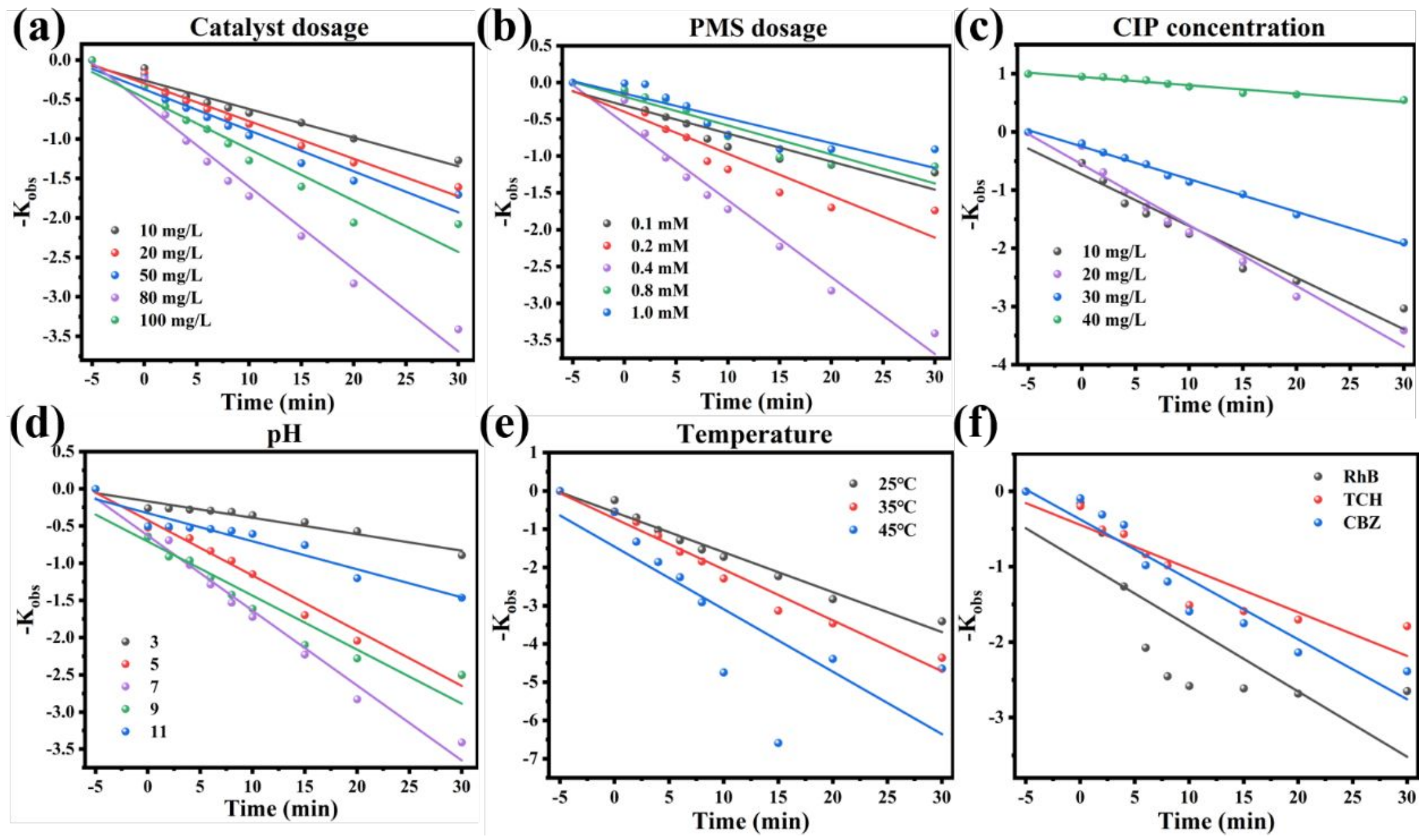

Fig. S7 Apparent rate constants corresponding to the effects of (a) catalyst dosage, (b) PMS dosage, (c) CIP concentration, and (d) initial pH, (e) Temperature, on the CIP degradation rate in the MIL@ZIF-67/SiC 1:2/PMS system. (f) Effect of other contaminant by the MIL@ZIF-67/SiC 1:2/PMS system.

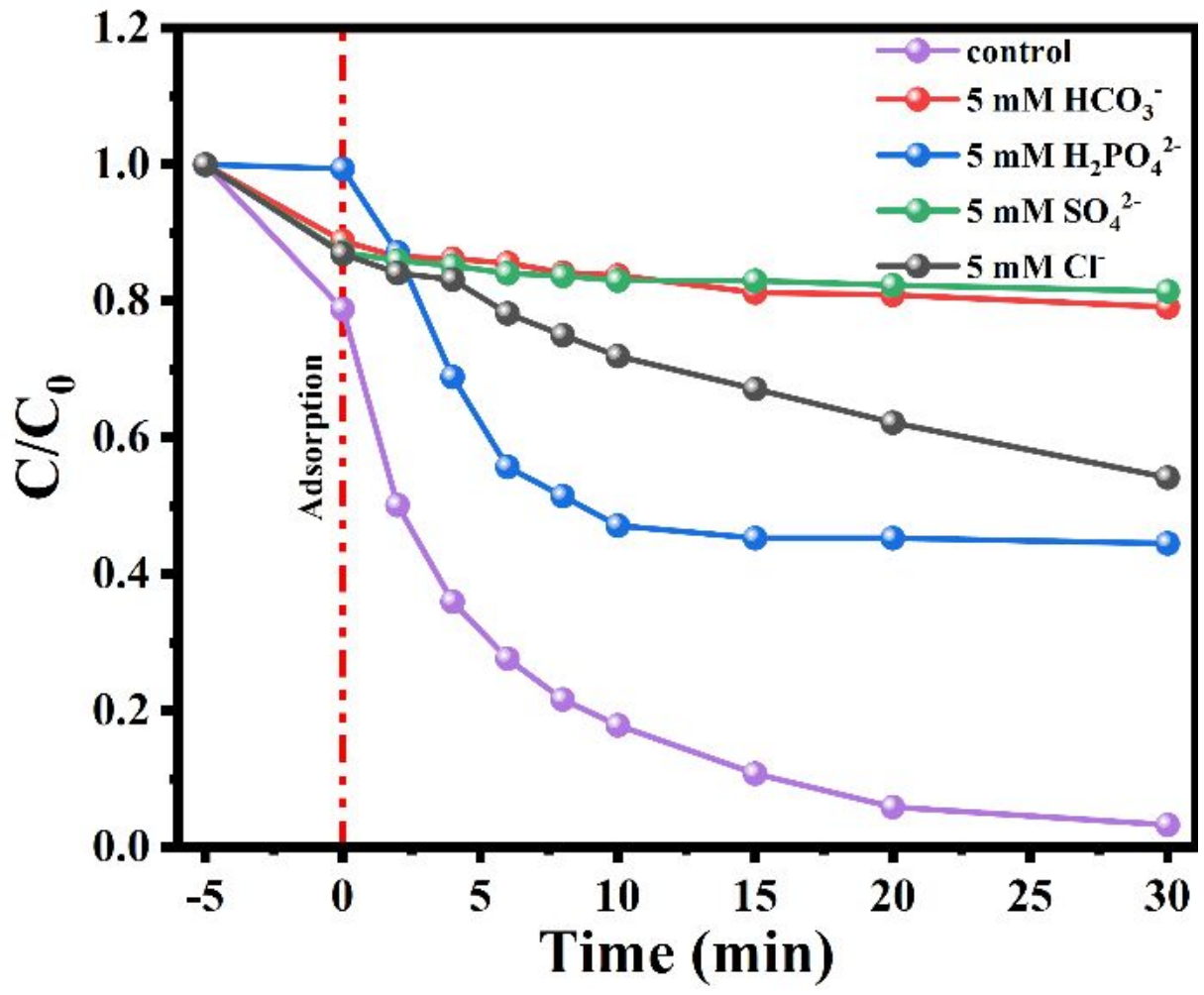

Fig. S8 Effect of co-existing ions on CIP degradation rate.

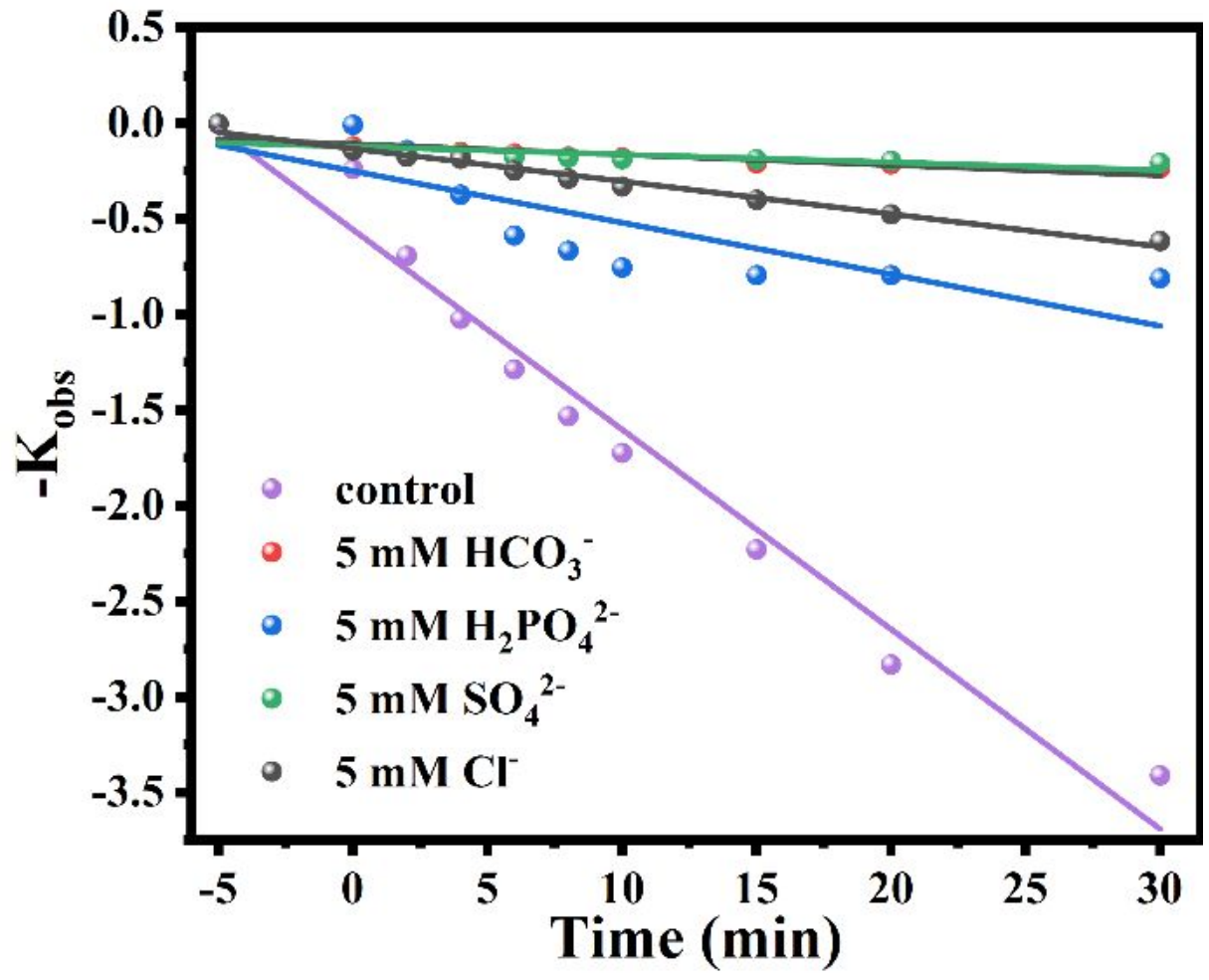

Fig. S9 Effect of coexisting ions on corresponding reaction rate constants.

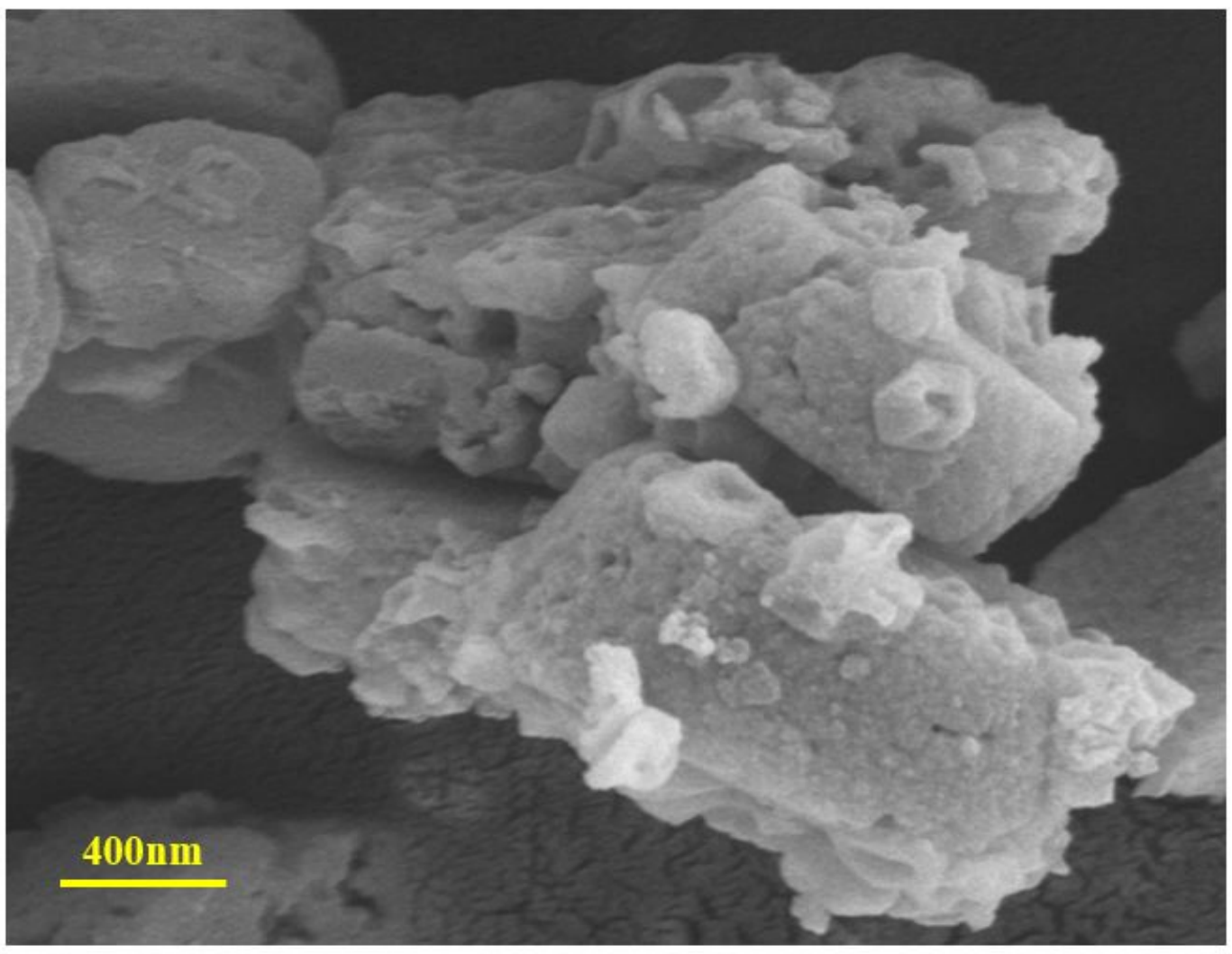

Fig. S10 SEM image of MIL@ZIF-67/SiC 1:2 after use.
